# GprC of the nematode-trapping fungus *Arthrobotrys flagrans* activates mitochondria and reprograms fungal cells for nematode hunting

**DOI:** 10.1038/s41564-024-01731-9

**Published:** 2024-06-14

**Authors:** Xiaodi Hu, David S. Hoffmann, Mai Wang, Lars Schuhmacher, Maria C. Stroe, Birgit Schreckenberger, Marcus Elstner, Reinhard Fischer

**Affiliations:** 1 Department of Microbiology, Institute for Applied Biosciences, Karlsruhe Institute of Technology (KIT) - South Campus, Karlsruhe, Germany; 2 Department of Theoretical Chemical Biology, Institute for Physical Chemistry, Karlsruhe Institute of Technology (KIT) - South Campus, Karlsruhe, Germany

**Keywords:** Fungal host response, Fungal genetics

## Abstract

Initiation of development requires differential gene expression and metabolic adaptations. Here we show in the nematode-trapping fungus, *Arthrobotrys flagrans*, that both are achieved through a dual-function G-protein-coupled receptor (GPCR). *A. flagrans* develops adhesive traps and recognizes its prey, *Caenorhabditis elegans*, through nematode-specific pheromones (ascarosides). Gene-expression analyses revealed that ascarosides activate the fungal GPCR, GprC, at the plasma membrane and together with the G-protein alpha subunit GasA, reprograms the cell. However, GprC and GasA also reside in mitochondria and boost respiration. This dual localization of GprC in *A. flagrans* resembles the localization of the cannabinoid receptor CB1 in humans. The *C. elegans* ascaroside-sensing GPCR, SRBC66 and GPCRs of many fungi are also predicted for dual localization, suggesting broad evolutionary conservation. An SRBC64/66-GprC chimaeric protein was functional in *A. flagrans*, and *C. elegans* SRBC64/66 and DAF38 share ascaroside-binding sites with the fungal GprC receptor, suggesting 400-million-year convergent evolution.

## Abstract (web summary)

A nematode-trapping fungus uses a dual-function G-protein-coupled receptor for perception of nematode-derived ascarosides for hunting *Caenorhabditis elegans*.

## Main

G-protein-coupled receptors (GPCRs) are widespread in all eukaryotes and represent the largest receptor family. Work on these proteins was honoured with the Nobel prize in 2012^1^. In humans, GPCRs are important drug targets^2^. GPCRs typically perceive external signals and transmit the signal from the plasma membrane through coupled G-proteins to different cellular actions^3^. Besides this canonical signalling, starting at the cytoplasmic membrane, localization of some receptors in other organelles in human cells suggests additional functions. One example is the human cannabinoid receptor CB1 which was found at mitochondria where it controls respiration^4^.

Lower eukaryotes, such as yeast or filamentous fungi, use GPCRs for nutrient sensing but also for communication before mating and interkingdom communication in pathogenic or symbiotic interactions^5,6^. Microbial interactions often rely on complex chemical signal exchange for recognition. In the case of predatory relationships, recognition should be followed by avoidance or defence reactions. Therefore, it is advantageous for the predator to sense prey-specific molecules with important functions for the prey because this will reduce the chance to escape recognition during evolution. However, such a dual function of molecules requires receptors for the same molecule in both organisms, predator and prey. In the case of nematode-trapping fungi, nematode-derived ascaroside pheromones serve this function^7,8^. They control many developmental processes in nematodes and are hijacked as signalling molecules by the fungal predator^9,10^. We study the predatory fungus *A. flagrans* (formerly *Duddingtonia flagrans*) and the model nematode *Caenorhabditis elegans*. *A. flagrans* produces adhesive trapping networks and overcomes the *C. elegans* defence also via small secreted proteins^8,10–13^.

## Results

### Three GPCRs and two G-protein alpha subunits of *A. flagrans* control trap formation

In *C. elegans*, eight ascaroside-sensing G-protein-dependent receptors (SRBC64, 66, SRG36, 37, DAF37, 38, SRX43 and 44) have been described, but in fungi, information on ascaroside-sensing receptors is lacking^14,15^. To identify GPCRs involved in the control of trap formation and thereby potentially in ascaroside sensing, we analysed the genome of *A. flagrans* by standard protein Blast and identified 14 putative receptor-encoding genes using *Aspergillus nidulans* and *Saccharomyces cerevisiae* GPCRs as baits. Due to similarities to characterized GPCRs, some proteins are not likely to sense ascarosides (Extended Data Table 1). Therefore, we focused our work on six candidates, GprA–F. Sequence analyses revealed a putative signal peptide only in GprC (first 30 amino acids at the N terminus) (https://ipsort.hgc.jp). In addition, GprC contains a putative mitochondrial targeting signal (MTS) cleaved after the 82nd amino acid (possibility of 98.41%) (https://ihg.helmholtz-munich.de/ihg/mitoprot.html). The probabilities for mitochondrial targeting were much lower for GprD, E and F (predicted to be cleaved at the 13th, 13th and 34th amino acids with possibilities of 2.58, 43.86 and 34.12%, respectively).

**Fig. 1:**
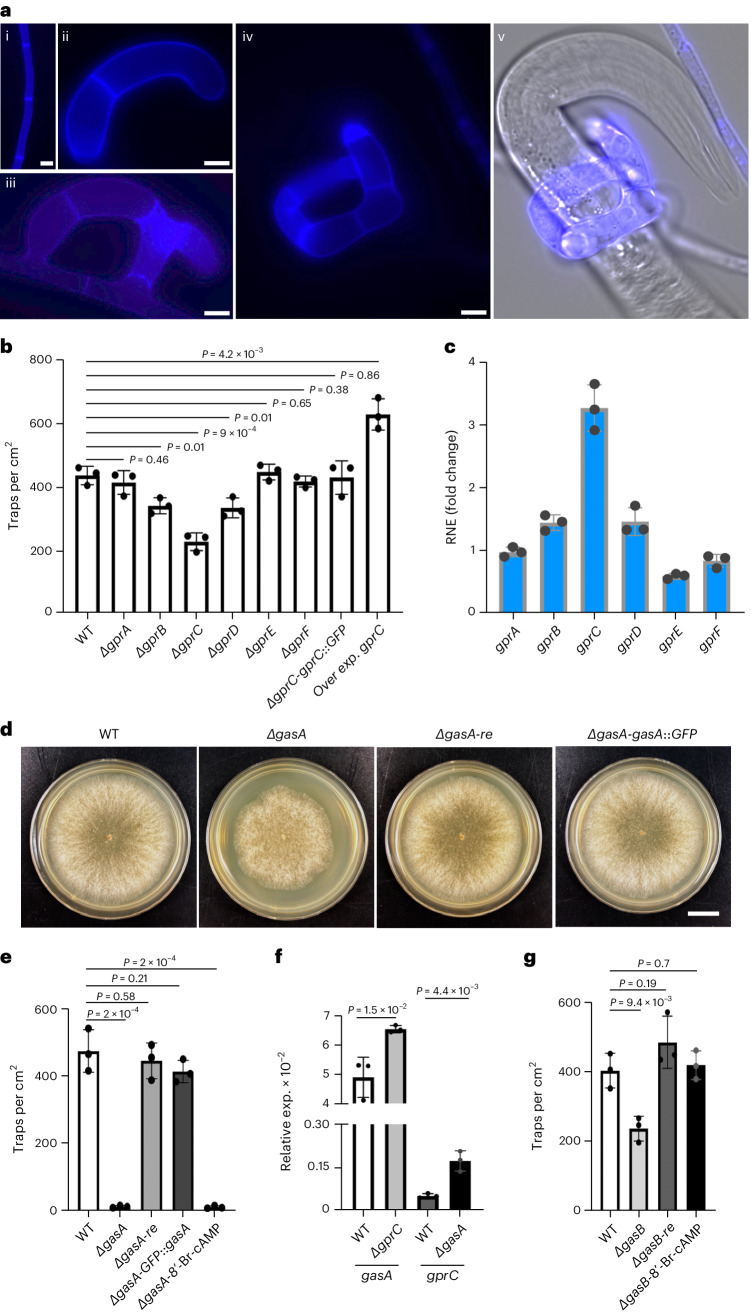
Analysis of the role of GPCR receptors and G-proteins in the control of trap formation in *A. flagrans.* **a**, Trap formation: (i) vegetative hypha, (ii and iii) different stages of trap formation, (iv and v) trap with immobilized *C. elegans*. The cell wall of the fungus was stained with calcofluor white. Scale bar, 5 µm. Microscopic images are representative of three independent repeats. **b**, Quantification of the number of traps in WT and six different GPCR receptor mutants, together with the recomplemented strain of the *gprC* mutant by C-terminally GFP-fused GprC under the native promoter and the *gprC-*overexpressing strain (mean ± s.d., *n* = 3 biological replicates; noted *P* values are from two-sided unpaired Student’s *t*-test compared to WT). **c**, Comparison of the expression of the six GPCR-encoding genes in control hyphae and hyphae induced with nematodes by RT–qPCR. The *y* axis shows the fold change of the expression of the *gprA*, *gprB*, *gprC*, *gprD*, *gprE* and *gprF* genes in the WT strain induced by nematodes for 6 h relative to 0 h. Each dot indicates one replicate. Bars and error bars indicate means ± s.d. of 3 biological replicates. The gamma actin orthologue DFL_002353 was used for normalization. **d**, Colonies of WT, the *gasA*-deletion strain and the *gasA-*deletion strain recomplemented with GasA (re) or GFP-GasA expressed from the native promoter. Scale bar, 1 cm. **e**,**g**, Quantification of trap formation of the indicated strains. 8’-Br-cAMP was used at a concentration of 5 mM. Data are expressed as mean ± s.d. (*n* = 3 biological replicates). *P* values were determined using a two-sided unpaired *t*-test compared to WT. **f**, Expression analysis of *gasA* and *gprC* in the indicated strains by RT–qPCR (mean ± s.d., *n* = 3 biological replicates; noted *P* values are from a two-sided unpaired Student’s *t*-test compared to WT). Source data

**Extended Data Fig. 1:**
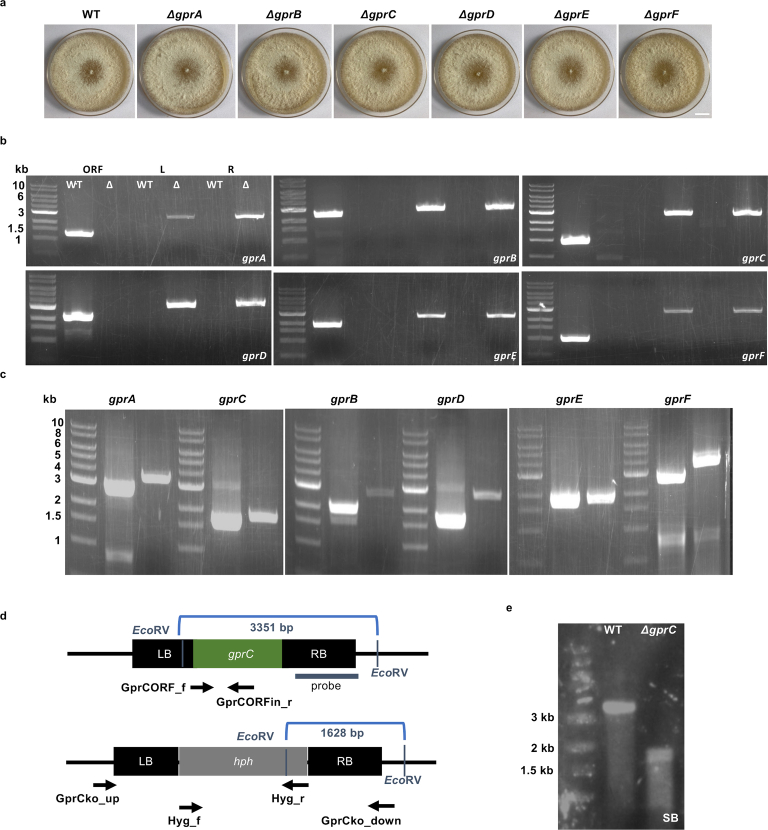
Deletion of six GPCR genes in *A. flagrans.* **(a)** Colonies of WT and *gprA-F*-deletion strains. Scale bar, 1 cm. **(b)** Confirmation of mutants by PCR. ORF fragments were amplified with primers of gprX ORF_f and ORF_r (or ORFin_r), L (left flanking borders) with ko_up_for (or ex_for) and hyg_rev, and R (right flanking borders) with hyg_for and ko_down_rev (or ex_rev). PCR gel pictures are representative of three independent repeats. **(c)** Confirmation of mutants by PCR. Left lanes show bands from the WT strain and right ones from the corresponding mutants. Fragments of *gprA* were amplified with primers of gprA_ex_for and gprA_ex_rev, *gprB* with gprB_ex_for and gprB_orf_rev or hyg_rev, *gprC* with gprCORF_for, gprCORF_rev, hyg_for and hyg_rev, *gprD* with gprD_ex_for and gprD_orf_rev or hyg_rev, *gprE* with gprEORF_for, gprEORF_rev, hyg_for and hyg_rev, *gprF* with gprF_ex_for and gprF_ex_rev. PCR gel pictures are representative of three independent repeats. **(d)** Scheme of the deletion strategy of *gprC*. Arrows and the dark blue line indicate the used oligonucleotides for PCR and the probe for the Southern blot. **(e)** Southern blot analysis of WT and the ∆*gprC* mutant. Genomic DNA was digested with *Eco*RV and the blot hybridized with the probe indicated in **(d)**. The blot is representative of three independent repeats.

To functionally characterize the six GPCRs, we deleted the corresponding genes, *gprA–F* (Extended Data Fig. 1a–e). The most drastic reduction in trap numbers occurred after deletion of *gprC*, whereas deletion of *gprB* or *gprD* had smaller effects (Fig. 1a,b). Another indication for a role in a signalling process can be their expression at the gene level. This was studied in starved fungal mycelia before and after exposure to nematodes by quantitative PCR with reverse transcription (RT–qPCR). Significant upregulation in the presence of nematodes was found in the case of *gprC* (Fig. 1c). Furthermore, trap number was significantly increased after overexpression of the *gprC* gene using the *oliC* promoter from *A. nidulans* (Fig. 1b). These lines of evidence strongly suggest a function of GprC, and possibly GprB and D, in nematode (ascaroside) sensing.

**Extended Data Fig. 2:**
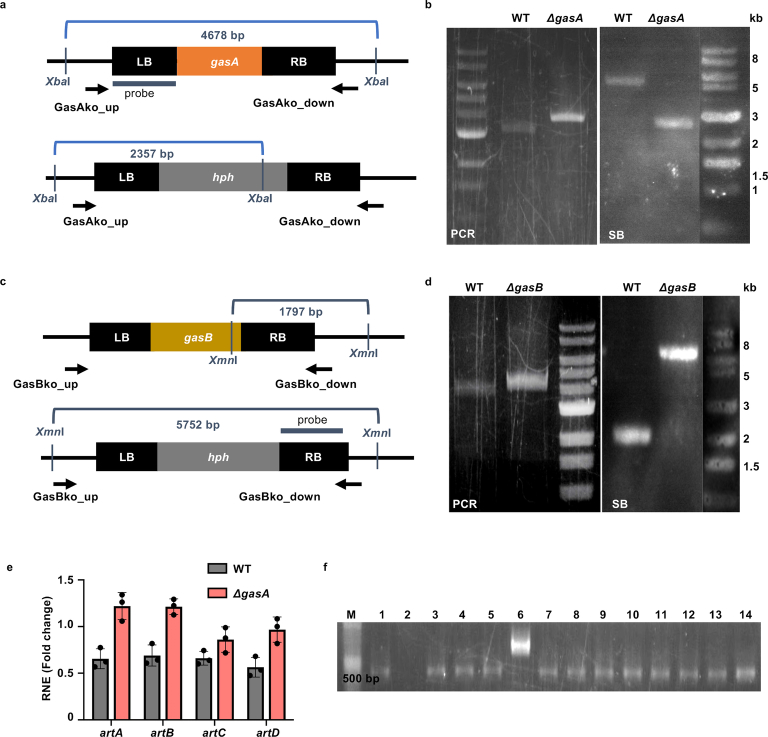
Deletion of *gasA* and expression analysis. **(a)** Scheme of the deletion strategy of *gasA*. **(b)** Confirmation of *gasA* deletion by PCR and Southern blot. The fragments after digestion of the genomic DNA with *Xba*I and the probe are indicated in **(a)**. PCR gel picture and blot are representative of three independent repeats. **(c)** Scheme of the deletion strategy of *gasB*. **(d)** Confirmation of *gasB* deletion by PCR and Southern blot. The expected fragments and the probe are indicated in **(c)**. PCR gel picture and blot are representative of three independent repeats. **(e)** The expression changes of the *artA*-gene cluster in nematode-induced hyphae of WT and the *gasA*-deletion strain compared with un−induced hyphae. RT–qPCR results are displayed as *fold change*. Bars and error bars indicate means ± s.d. of three biological replicates. The gamma actin orthologue DFL_002353 was used for normalization. **(f)** Confirmation of *gprC* re−complemented strains by RT–PCR and the amplified fragments of 374 bp showing the expression of various versions of mutated *gprC* genes. Strains are from left to right: (1) WT, (2) *gprC* mutant, (3) *gprC* re-complemented strain and the chimeric protein re-complemented strains (4) *SRBC64-gprC-re*, (5) *SRBC64*^*N2.60A*^*-gprC-re*, (6) full length *SRBC66-re* with an unspecific band, (7) *SRBC66-gprC-re*, (8) *DAF37-gprC-re*, (9) *DAF38-gprC-re*, (10) *Octr-1-gprC-re*, and (11) mutated *gprC*^*R6.45A*^*-re*, (12) *gprC*^*S2.41A*^*-re*, (13) *gprC*^*N2.57A*^*-re* and (14) *gprC*^*N2.53A*^*-re*. The PCR gel picture is representative of three independent repeats.

Typically, GPCR-dependent signalling cascades consist of downstream G-proteins connected to other signalling modules such as MAP kinase cascades. Ultimately, activation of the signalling cascades leads to differential gene regulation. To identify putative G-proteins downstream of GprC, we studied the role of three G-protein alpha subunits of *gasA* (*dfl_009501*), *gasB* (*dfl_000801*) and *gasC* (*dfl_009358*) in trap morphogenesis. Deletion of *gasA* resulted in complete loss of trap formation, and recomplementation with a wild-type (WT) copy restored the WT phenotype (Fig. 1d,e and Extended Data Fig. 2a,b). An N-terminally GFP-tagged GasA version also recomplemented the mutant phenotype, suggesting that the fusion protein (used for localization in experiments below) is biologically functional (Fig. 1e). Deletion of *gprC* caused upregulation of *gasA*, and deletion of *gasA* resulted in *gprC* induction (Fig. 1f). Previously, it was shown that the MAP kinases Fus3, Slt2 and Hog1 are involved in trap formation and are probably downstream of a G-protein^16–18^. Another possibility for G-protein-dependent signalling is through changes of the cAMP level^19^. To distinguish between the two possibilities, MAP kinase versus cAMP signalling by GprC and GasA, we tried to rescue the *gasA-*mutant phenotype by adding the cAMP analogue 8’-Br-cAMP. No stimulation of trap formation in the mutant was observed (Fig. 1e). The analogue did not influence trap formation in WT. This result suggests that GprC–GasA channel the signal into MAP kinase pathways or use other signalling cascades. Deletion of *gasB* also affected trap initiation (Fig. 1g and Extended Data Fig. 2c,d), but *gasC* appeared to play no role (data not shown). The *gasB-*deletion phenotype could be rescued by 8’-Br-cAMP, suggesting that GasB uses the cAMP pathway for signal transduction (Fig. 1g).

### GprC interacts with GasA at the cytoplasmic membrane and at mitochondria

**Fig. 2:**
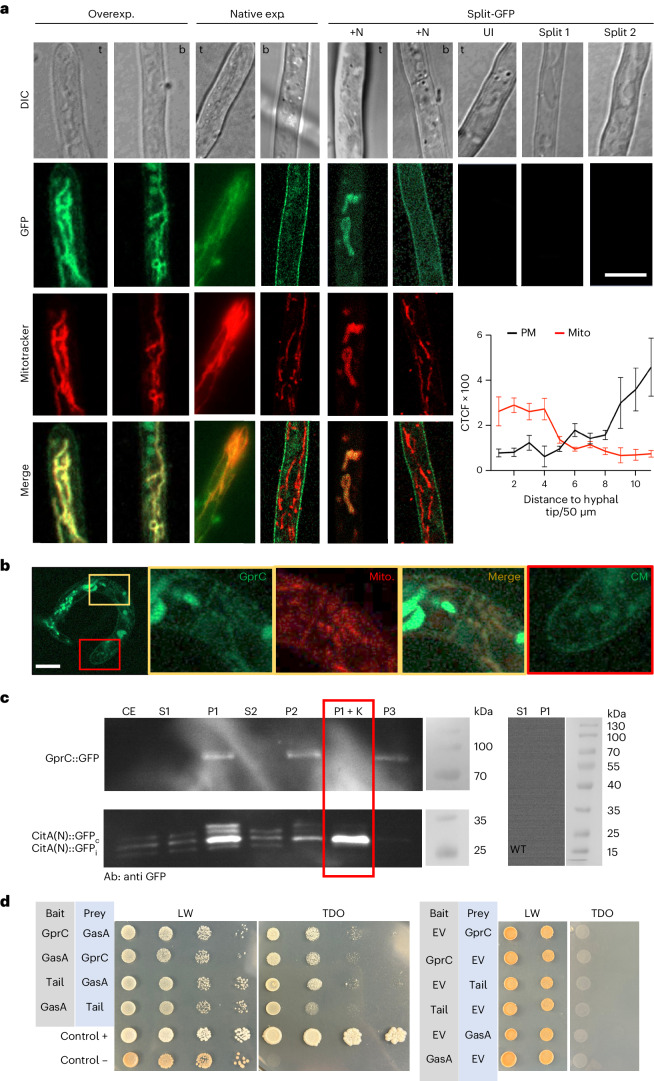
GprC and GasA reside and interact in the cytoplasmic membrane and in mitochondria. **a**, Left: localization of GprC after overexpression (Overexp.) or with native expression (Native exp.). Mitochondria were stained with mitotracker. Left pictures show hyphal tips (t) and right pictures areas away from the tip (b). Right: visualization of GprC–GasA interaction. *gprC* and *gasA* were expressed from their own promoters. The left pictures show a hyphal tip (t) and the hypha further back (b) after induction with nematodes (+N). The right pictures show uninduced hyphae (UI) or control hyphae with only one of the constructs expressed (Split 1 and Split 2). Scale bar, 5 µm. Bottom right: quantification of GprC-GFP in a hypha from tip to back (550 µm). The hypha was divided into 11 sections and fluorescence quantified at 3 to 7 places in each section. The mean of the values is displayed (mean ± s.d., *n* = 3–7 biological replicates). **b**, Localization of GprC-GFP in a trap. The yellow-boxed area shows mitochondrial and the red box cytoplasmic membrane localization. Scale bar, 5 µm. Microscopic images are representative of three independent repeats. **c**, Cell fractionation of WT, GprC-GFP- and CitA(N)-GFP-expressing strains. Crude extract (CE), supernatants (S1 and 2), pellets (P1, 2 and 3) and the digested pellet 1 by proteinase K (P1 + K) (red box) were analysed with an anti-GFP antibody. Crude extract of WT was used as negative control. Blots are representative of three independent repeats. **d**, Left: interaction of GprC or the tail of GprC (111 amino acids) with GasA confirmed with Y2H analysis. LW, SD medium without leucine and tryptophan; TDO, triple dropout medium (SD medium without leucine, tryptophan and histidine). Right: single gene controls . Source data

Next, we tested the hypothesis that GprC and GasA interact at the protein level. First, we aimed at localizing GprC in *A. flagrans* and tagged GprC at its C terminus. GprC-GFP was expressed using the constitutively active and quite strong *oliC* promoter. The fusion protein localized at filamentous structures inside the fungal compartments and not, or at least not visibly, at the cytoplasmic membrane. Costaining with mitotracker revealed that the intracellular structures were mitochondria^20^ (Fig. 2a). The tagged protein was able to complement the *gprC-*deletion phenotype, suggesting that the fluorescent protein tag does not interfere with the function (Fig. 1b). Mitochondrial morphology was not obviously different from mitochondria in WT despite the overexpression of GprC. When the same GprC-GFP construct was expressed from its natural promoter, the localization pattern was more complex. Whereas the protein appeared at mitochondria in hyphal tips, the protein localized at the cytoplasmic membrane in compartments away from the tip. To investigate whether dual localization reflects dual action of the receptor, we tested for interaction of GprC with GasA and used a bifluorescent complementation system (split GFP). GprC was tagged at the C terminus with the C-terminal half of GFP. GasA was tagged N terminally with the N-terminal half of GFP. Neither construct alone resulted in fluorescent *A. flagrans* strains, but the combination of the two highlighted the plasma membrane and mitochondria in the same way as the GprC-GFP fusion protein when nematodes were present (Fig. 2a). In traps, GprC was found at the cytoplasmic membrane and in mitochondria, but a gradient in the localization was not observed. In addition, traps contained some autofluorescent signals observed in the GFP channel (Fig. 2b).

**Extended Data Fig. 3:**
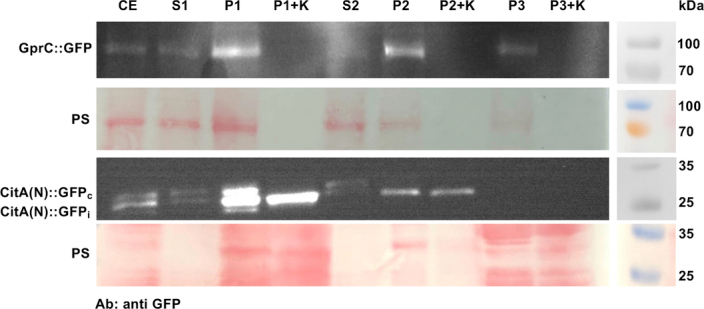
GprC resides in mitochondria and the cytoplasmic membrane. The GprC-GFP and CitA(N)-GFP expressing strains were cultivated overnight in PDB medium and harvested for cellular fractionation. Crude extract (CE), supernatants (S1 & 2), pellets (P1, 2 & 3) and the proteinase K digested pellets (P1 + K, P2 + K and P3 + K) were analyzed in a Western blot using an anti-GFP antibody. GprC-GFP is about 90 kDa. CitA(N)-GFP appeared as cytoplasmic fusion protein (around 33.2 kDa) and a shorter imported version (around 30.2 kDa) in the mitochondria which remained after protease K digestion, plus a smaller degradation product. Both membranes were stained with Ponceau S (PS) before the Western blot. Blots are representative of three independent repeats.

To confirm the obtained localization and interaction results, we isolated mitochondria from *A. flagrans* expressing GprC-GFP and tested them for the presence of GprC (Fig. 2c and Extended Data Fig. 3)^21^. The protein amount was normalized to the volume of the original protein extract. After fractionation, mitochondria were enriched in pellets 1 and 2 (P1, 400*g*; P2, 11,000*g*) and the cytoplasmic membrane in pellet 3 (P3, 100,000*g*). GprC-GFP (~90 kDa) was detected in the mitochondrial and the plasma membrane fractionation. The fusion protein of all three pellets was completely degraded after digestion with proteinase K (P1, 2 and 3 + K), suggesting that the mitochondrial GprC protein resides in the outer mitochondrial membrane. As a positive marker for mitochondria, we targeted GFP to the mitochondrial matrix using the N-terminal region (40 amino acids) of citrate synthase (CitA), established in *A. nidulans*^20^. The CitA(N)-GFP fusion protein has a molecular mass of 33.2 kDa and after import, 30.2 kDa. Both bands were visible, plus a degradation product (in P1). After treatment of proteinase K, the 30.2 kDa band remained, whereas the non-imported fusion protein (33.2 kDa) band disappeared. This showed that the cytoplasmic protein variant was digested (CitA(N)::GFP_c_) and the mitochondrial one (CitA(N)::GFP_i_) was protected.

GprC–GasA protein interaction was confirmed using a yeast two-hybrid (Y2H) assay. The C-terminal tail (111 amino acids) of GprC appeared to interact slightly stronger with GasA than full-length GprC (Fig. 2d).

### GprC–GasA signalling controls gene expression and mitochondrial respiration

**Fig. 3:**
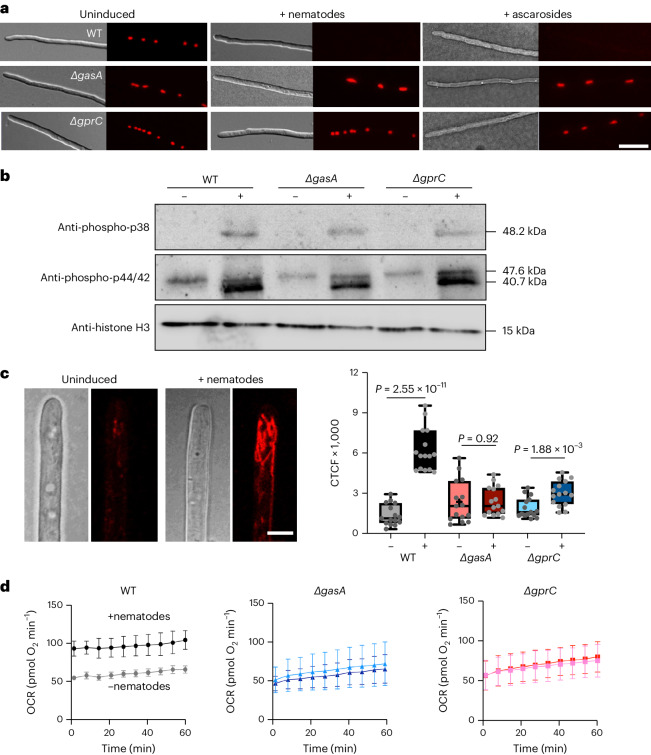
GprC and GasA-dependent signalling. **a**, Expression of *artA* in a promoter–reporter assay. Microscopy of hyphae, uninduced or induced with nematodes or ascaroside #18, of WT and a ∆*gasA* and a ∆*gprC* mutant. Scale bar, 10 µm. Microscopic images are representative of three independent repeats. **b**, Phosphorylation analysis of HogA, MakA and MakB (molecular masses of 48.2, 47.6 and 40.7 kDa, respectively) and the histone H3 control (15 kDa) in control (−) and induced (+) mycelia. Conidia (10^6^) were grown on cellophane on LNA plates for 5 days before 10,000 N2 nematodes were added for 3 h followed by fungal protein extraction. Protein (35 µg) were analysed using western blot. Blots are representative of three independent repeats. **c**, Visualization and quantification of the ROS signals in mitochondria of uninduced and nematodes-induced hyphae stained by CellROX orange. Scale bar, 3 µm. Five fluorescent signals were quantified with Fiji software in three hyphae (*n* = 15 from three biological replicates). Boxplots show median (centre line), 25th to 75th percentiles (box limits), the minimum and maximum values (whiskers) and individual values as points superimposed on the graph. For *P* values, a two-sided unpaired Student’s *t*-test was performed to compare uninduced and induced hyphae. **d**, Oxygen consumption rate of mycelia of indicated strains grown in liquid LN medium. Curves in light colours (grey, light blue and pink) indicate uninduced mycelia, while dark colours (black, dark blue and dark red) represent nematode-induced mycelia (mean ± s.d., *n* = 3 biological replicates). Source data

To understand which genes may be controlled by *C. elegans*, the interkingdom signalling process between fungus and nematode has to be understood. *C. elegans* is lured into the fungal mycelium and into the traps by 6-methyl-salicylic acid (6-MSA) and small, volatile molecules that mimic a sexual partner and/or food^8,22,23^. In addition, *A. flagrans* produces polyketide derivatives, arthrosporols and 6-MSA as inhibitors of trap formation in the absence of nematodes^8,24^. If nematodes are present and ascarosides reach a certain threshold concentration, arthrosporol and 6-MSA productions are inhibited and traps are formed. Hence, ascarosides repress the expression of the polyketide synthase gene, *artA*, required for arthrosporol and 6-MSA biosynthesis. The expression of the *artA-*cluster genes was quantified by RT–qPCR (Extended Data Fig. 2e). The presence of nematodes reduced the expression level of *artA–D* slightly, in comparison with WT without nematodes. In the *gasA*-deletion strain, the absence or presence of nematodes did not affect the expression levels. The differences were not very pronounced because in older hyphal compartments, the *artA*-gene cluster is again activated to inhibit excessive trap formation^8^. To obtain more convincing results, the expression of *artA* was studied in a promoter–reporter assay which allows for cellular resolution of expression. The *artA* promoter was fused to the mCherry- and a histone-encoding gene. In WT, strong mCherry signals were observed in nuclei of vegetative hyphae. After the addition of nematodes or ascaroside #18, the signals disappeared (Fig. 3a). We used ascaroside #18 because it is commercially available and was also shown to induce trap formation^25^. In the *gasA-* and the *gprC-*deletion strains, the *artA* promoter did not respond to *C. elegans* or ascaroside #18. These results suggest a canonical function of GprC and GasA. Next, we tested whether MAP kinases would be phosphorylated and thereby activated. The characterized MakB (DFL_000344), which participates in hyphal fusion, and homologues of MakA (Slt2) (DFL_005546) and HogA (DFL_000806) were analysed for their phosphorylation status with antibodies against the phospho-p38 MAPK and the phospho-p44/42 MAPK^17,18,26^. Same amounts of extracted protein of uninduced and induced mycelia from WT and the *gasA-* and the *gprC-*deletion strains were processed for western blotting. After 3 h of induction by nematodes, HogA and MakB (48.2 and 40.7 kDa) were phosphorylated in WT and mutant strains, which was not seen in uninduced hyphae (Fig. 3b). A weak phosphorylation level of MakA (47.6 kDa) was observed in all samples. This result suggests that the GprC–GasA signalling is independent of the three MAP kinases.

Mitochondrial localization of GprC suggested direct effects on respiration. This hypothesis was tested by staining the fungal hyphae for the presence of reactive oxygen species (ROS) (Fig. 3c). ROS are a byproduct of respiration. Growing hyphal tips showed weak fluorescence of mitochondria, which increased when the hyphae were exposed to *C. elegans*. In the absence of GprC or GasA, the increase in fluorescence was much smaller and not significant. The fluorescent signal was very weak or not detectable in hyphal compartments away from the tip. These results suggest stimulation of respiration in mitochondria at the hyphal tip through GprC–GasA signalling. To further confirm this effect on mitochondria, we measured the oxygen consumption rates (OCR) of fungal hyphae and fungal hyphae incubated with *C. elegans*. Indeed, WT hyphae consumed more oxygen after induction with nematodes as compared with noninduced hyphae. The oxygen consumption rate was unaffected by nematodes in the ∆*gprC* or the ∆*gasA* mutant strains (Fig. 3d).

Taken together, the results suggest two functions of the GPCR protein and the G-protein alpha subunit at the cytoplasmic membrane and in mitochondria, respectively. Although there is currently no information about the cellular path for mitochondrial targeting, direct transfer from the ER membrane to the mitochondrial membrane is one possibility^27,28^.

### *A. flagrans* GprC and *C. elegans* SRBC64/66 share the ascaroside-binding site

**Fig. 4:**
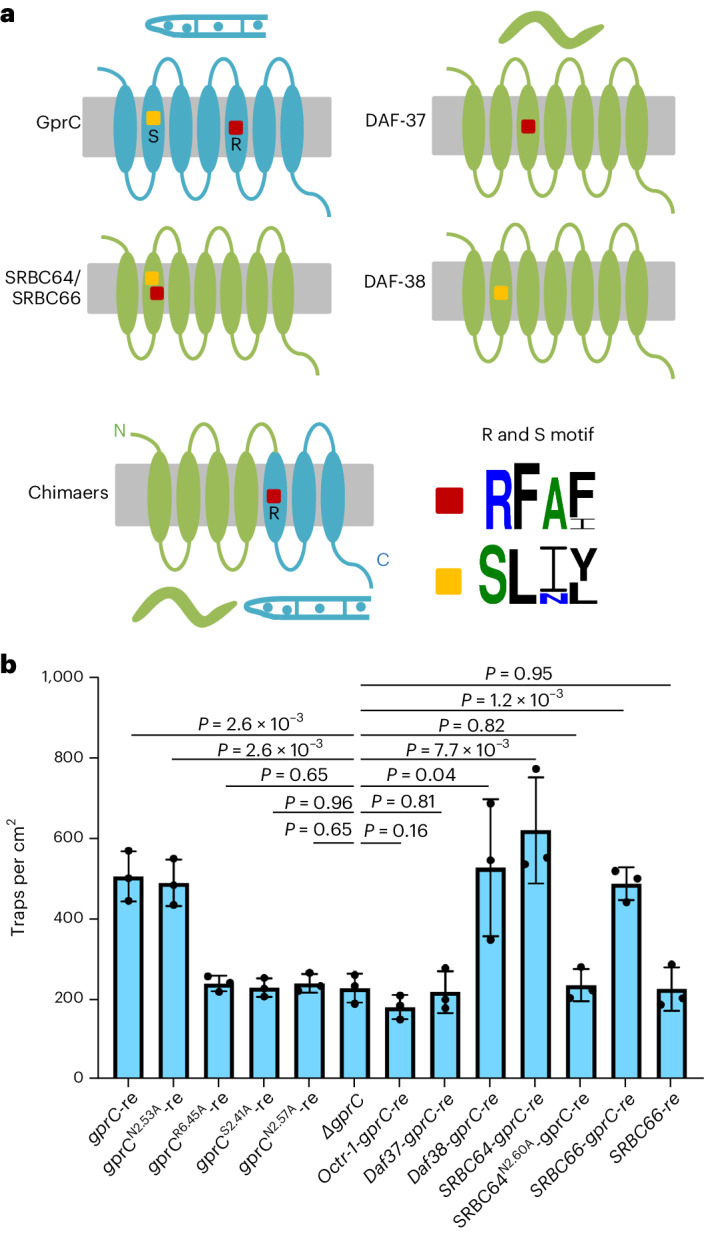
Structural and functional comparison of *A. flagrans* and *C. elegans* ascaroside-sensing GPCRs. **a**, Scheme of GprC, DAF37, DAF38, SRBC64/66 and a chimaeric protein. The conserved R and S motifs are indicated. **b**, Biological activity of *A. flagrans*–*C. elegans* chimaeric receptor proteins and of different *gprC* mutant alleles. The trap production of varied rescuing strains was compared to the *gprC* mutant strain. Strains are (from left to right): *gprC* recomplemented strain (1), recomplemented strains with mutated *gprC* genes (*gprC*^*N2.53A*^*-re* (2), *gprC*^*R6.45A*^*-re* (3), *gprC*^*S2.41A*^*-re* (4) and *gprC*^*N2.57A*^*-re* (5), the *gprC* mutant (6)), and the chimaeric protein recomplemented strains *Octr-1-gprC-re* (7), *DAF37-gprC-re* (8), *DAF38-gprC-re* (9) and *SRBC64-gprC-re* (10), mutated *SRNC64*^*N2.60A*^*-gprC-re* (11), *SRBC66-gprC-re* (12), and the strain *SRBC66-re* (13) including full length of nematode receptor SRBC66-encoding gene (mean ± s.d., *n* = 3 biological replicates; noted *P* values are from two-sided unpaired Student’s *t*-test compared to *gprC* mutant).

Next, we asked how the ascaroside-sensing GPCRs in *C. elegans* and *A. flagrans* could have evolved. The predatory lifestyle of these fungi dates back more than 400 million years, and fungi could have acquired ascaroside receptors from nematodes by horizonal gene transfer or by convergent evolution^29^. In *C. elegans*, SRBC64 recognizes ascaroside #1 but not ascaroside #5. Likewise, ascaroside #1 was more effective in trap induction in *A. oligospora* than ascaroside #5 (ref. ^7^). Therefore, we hypothesized that the fungal GPCR should be similar to *C. elegans* SRBC64/66. However, none of the 14 fungal GPCRs shares extended sequence similarities to SRBC64/66, ruling out horizontal gene transfer. Nevertheless, sequence comparisons of GprC and the ascaroside-sensing GPCRs of *C. elegans* revealed two short conserved sequence motifs (S and R), suggesting conservation of ascaroside binding and/or signalling (Fig. 4a). Next, we tested whether any of the *C. elegans* receptors could complement the lack of GprC in *A. flagrans*. Four candidates were successfully amplified from *C. elegans* complementary DNA, whereas we failed to clone SRG36/37 and SRX43/44. The four candidates, SRBC64/66 and Daf37/38 were expressed in *A. flagrans* under the control of the *gprC* promoter, but none of the transgenic strains recovered trap formation to WT levels. One reason for the failure of complementation could be specific downstream signalling components in the fungus and the nematode. Therefore, we constructed chimaeric proteins, always keeping the last three transmembrane helices to enable intracellular fungal signalling. The combination of the first four transmembrane (TM) helices of SRBC64/66 and DAF38, but neither DAF37 nor Octr-1 (a non-ascaroside receptor of *C. elegans* as a negative control)^30^, with the three fungal TM helices resulted in functional receptors (Fig. 4b). The expression of all GPCR genes (*gprC* variants, chimaeric version or *C. elegans* receptor genes) in *A. flagrans* was confirmed by RT–PCR (Extended Data Fig. 2f). Comparison of these results with the distribution of the S and the R motifs in the sequences revealed the need for both motifs in the receptor. Interestingly, SRBC66 (probability of 82.92% for mitochondrial localization) and SRBC64 (with lower probability of 29.71% for mitochondrial localization) were also predicted by mitoprot and ipsort for dual localization at the cytoplasmic membrane and in mitochondria. This was not the case for DAF37 and DAF38.

**Fig. 5:**
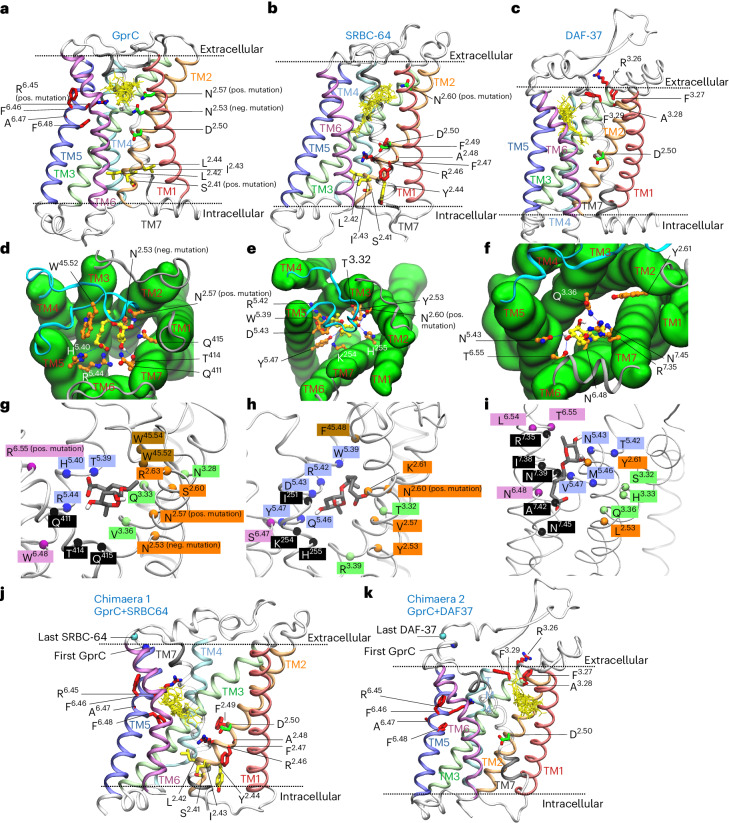
Docking models for different ascarosides and different GPCRs. **a**–**c**, Twenty-five binding poses of ascarosides #1, #2, #3, #5 and #18 (yellow) docked into different receptor models. A highly conserved Asp residue in TM2 is shown in green. Extracellular side on top, intracellular side at the bottom. **d**–**f**, Binding pose of ascaroside #1 from extracellular view showing conical arrangement of the receptor. Extracellular loop 2 is shown in blue. **g**–**i**, Binding pose of ascaroside #1 with all residues within 3.5 Å not considering glycine, proline or backbone atoms. **j**,**k**, Twenty-five binding poses of the five ascarosides (yellow) docked into chimaeric receptors.

To further validate the hypothesis that the receptors in *A. flagrans* and in *C. elegans* evolved by convergence, we performed docking studies on receptor models of GprC, SRBC64 and DAF37 to identify and compare the ascaroside-binding sites in the fungal and nematode receptors. TMs 2 and 3 in DAF37 are longer and more tilted than the corresponding helices in the other two receptors. The top five binding poses of ascarosides #1, #2, #3, #5 and #18 were analysed using Autodock Vina^31,32^, resulting in a total of 25 different binding poses (Fig. 5a–c and Extended Data Table 2).

In addition, we tested the binding affinity of the putative ligands, glucose and sucrose, as many Gpr1 homologues are known to be nutrient sensors^33^. Models of GprB and GprD were also used. The estimated binding energies of the best predicted poses are shown in Extended Data Table 2. Missing dynamics, such as loop movement or induced fit mechanism, may affect the result. Therefore, it is crucial to avoid overinterpretation and instead prioritize the various binding poses. All putative ligands occupied the classical orthosteric binding sites of GPCRs, with TMs 1 and 4 not involved in binding. Even though there are seven TM domains (Fig. 5a), only five of them work actively in binding ligands (Extended Data Table 3), which is typical for GPCRs^34^. The loop connecting TMs 4 and 5 is called extracellular loop 2 (ECL2) and has been shown to be involved in ligand binding in many GPCRs^34–36^. The ECL2 in GprC and SRBC64 coordinated ascarosides, whereas in DAF37 it did not bind any of the 20 poses and is much longer than in the other two receptors.

SRBC64 and DAF37 are members of the solo families srbc and srw, respectively^37^. To compare GPCRs across different families, several generic residue numbering schemes have been introduced^38^. The Ballesteros–Weinstein numbering scheme^39^ uses the most conserved residue for each TM helix individually and denotes it as number 50. For example, Pro2.53 denotes a proline residue in TM2, which is three residues behind the most conserved residue. In the three receptors, we found Asp2.50 which is highly conserved between different GPCRs in many organisms (Fig. 5a–c). In most GPCRs, the highly conserved residue in TM6 is a proline, which is present in GprC and SRBC64. DAF37 has a tryptophan instead. Regarding the most conserved residues, the length of ECL2 and the shape of TMs 2 and 3, the two receptors GprC and SRBC64 are more similar to each other than to DAF37.

By counting all receptor residues located within 3.5 Å of one of the 20 binding poses, we obtained an ensemble of coordinating residues for all three receptors (Extended Data Table 3). Due to the steric hindrance of the conical receptor, a binding pocket on the intracellular side is unlikely (Fig. 5d–f). To test the predicted binding sites for function, we performed mutagenesis studies on certain amino acids. In GprC, two asparagines in TM2 were selected, separated by a helical loop. Mutation of the more intracellular N2.53 to alanine showed no effect on receptor function (neg. mutation), whereas mutation of N2.57 resulted in loss of function (pos. mutation), leading us to conclude that the orthosteric binding site on the extracellular side is occupied by ascarosides in GprC (Figs. 4b and 5a). In SRBC64, the asparagine N2.60 affected the function of the receptor after mutation, confirming the extracellular orthosteric binding site for SRBC64 (Fig. 5b). To compare the binding pockets, we looked at similar residues in the same relative position in a TM. Apart from aromatic residues in ECL2, a polar residue in 2.60 and a basic residue in 2.61 or 2.63, no motifs of the same chemical nature were observed in the same position (Fig. 5g–i).

On the other hand, the R-motif is present in all three receptors. In GprC, the arginine R6.45 points to the binding site, a mutation to alanine leads to a drastic drop in binding affinity (Extended Data Table 2) and it has been experimentally confirmed to be essential for receptor function (Fig. 4b). In SRBC64 and DAF37, the R-motif is not involved in binding (Fig. 5a–c). The S-motif is only present in GprC and in SRBC64. In fact, in TM2 it is in the exact same position (S2.41). It is far from the binding site but has been experimentally confirmed to be essential for function (Fig. 4b). In the model of the chimaeric protein DAF37-GprC, which did not rescue the GprC function, the S-motif is missing, and a completely different binding site between the TMs 1, 2 and 7 is occupied. The chimaeric receptor SRBC64-GprC coordinated ascarosides between TM helices 3, 5, 6 and 7 (Fig. 5j,k). Since the S-motif is in the same relative position (S2.41) in the rescued chimaeric receptor, we hypothesize that it is essential for signalling.

## Discussion

**Fig. 6:**
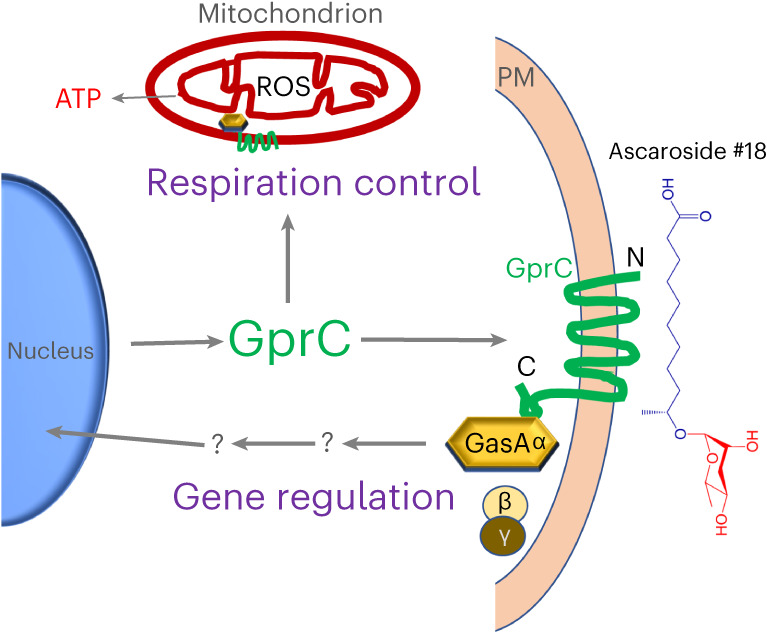
Model of GprC and GasA functioning at the cytoplasmic membrane and in mitochondria. GprC and GasA reside at the cytoplasmic membrane, interact with nematode-derived ascarosides and transmit the signal to the nucleus for gene regulation. In addition, both proteins localize at mitochondria for respiration control.

We show that a G-protein-coupled receptor protein of *A. flagrans* exhibits dual localization and function (Fig. 6). Whether both localizations are also required for the fungal–nematode interaction, however, remains to be determined. Although there are few examples for GPCR localizations in endosomes^40^ and mitochondria^41^, the functions of the proteins at the different places are not yet well understood^42^. Our results show that this property of GPCRs is conserved in evolution from fungi to humans and appears to be of much greater importance than so far anticipated. GPCR-dependent signalling cascades have been broadly studied in fungi because they are implicated in many environmental cues and are often crucial for host interactions in organisms ranging from plants to nematodes to humans. Our discovery of a dual-function GPCR in *A. flagrans* led us to analyse several GPCRs of other fungi. We identified GPCRs with predicted dual localization using the mitoprot server in the ascomycetes *A. nidulans* (GprH, 74% probability for mitochondrial localization)*, Aspergillus fumigatus* and *Metharhizium anisopliae* as well as in the basidiomycetes *Cryptococcus neoformans* and *Ustilago maydis*. In nerve and striated muscle cells, cannabinoid receptors inhibit respiration of mitochondria^4,43^. The effect of GPCR activation in mitochondria of *A. flagrans* appears to be opposite and respiration is activated. It will be the challenge of future research to unravel the connection between GPCR signalling and the respiratory chain and its function(s) in fungal growth and pathogenicity.

## Methods

### Strains and culture conditions

*A. flagrans* (CBS 349.94) was obtained from the CBS-KNAW culture collection (Westerdijk Institute, the Netherlands) and cultured at 28 °C on potato dextrose agar (PDA) for normal growth and on low-nutrient agar (LNA) (1 g l^−1^ KCl, 0.2 g MgSO_4_·7H_2_O, 0.4 mg MnSO_4_·4H_2_O, 0.88 mg ZnSO_4_·7H_2_O, 3 mg FeCl_3_·6H_2_O, 10 g agar, pH 5.5) for fungal starvation. N2 *C. elegans* was obtained from Prof. Dr Ralf Baumeister (University of Freiburg) and used as the WT. Standard cultivation and synchronization methods were used for *C. elegans* (10.1895/wormbook.1.101.1) and *S. cerevisiae*. All strains are listed in Supplementary Table 1 and Extended Data Table 4.

### Protoplast transformation of *A. flagrans*

*A. flagrans* was cultured on a 9 cm PDA Petri dish at 28 °C for 7 days. Mycelia were scratched off the agar and inoculated in 150 ml PDB medium and incubated for 24 h at 28 °C. The mycelium was collected and washed with MN solution (0.3 mol l^−1^ MgSO_4_, 0.3 mol l^−1^ NaCl) and ~0.5 g of mycelia (wet weight) was collected and suspended in 5 ml MN buffer containing 4 mg ml^−1^ kitalase (Fujifilm Wako Chemicals) and 20 mg ml^−1^ VinoTaste Pro (Novozymes), followed by incubation at 30 °C for 2 h. Quality of protoplasts was checked microscopically. Subsequently, undigested mycelia were removed by filtering the protoplast through two layers of miracloth tissue. Protoplasts were precipitated at 2,400*g* for 15 min. After carefully removing the supernatant, protoplasts were washed with 50 ml KTC buffer (1.2 mol l^−1^ KCl, 10 mmol l^−1^ Tris-HCl pH 7.5 and 50 mmol l^−1^ CaCl_2_) and the resulting pellet was resuspended in 500 μl KTC solution. For transformation, 100 µl protoplast suspension (2 × 10^7^) were mixed with 3 μg DNA and incubated for 2 min on ice. Then, 1 ml of PTC (10 mmol l^−1^ Tris-HCl pH 7.5, 50 mmol l^−1^ CaCl_2_, 60% w/v polyethylene glycol 6000) was added and incubated at room temperature for 20 min. PDSSA (10 ml; 24 g l^−1^ potato dextrose broth, 0.6 mol l^−1^ sucrose, 0.3 g l^−1^ peptone, 0.3 g l^−1^ yeast extract, 8 g l^−1^ agar) was added to the transformation mixture and the mixture poured onto PDA plates supplemented with 100 μg ml^−1^ hygromycin-B or 150 μg ml^−1^ geneticin (G418) and incubated at 28 °C for 5–7 days.

### Plasmid construction

Q5 High-Fidelity DNA polymerase for PCR and restriction enzymes were purchased from New England Biolabs. Plasmids were assembled using the NEBuilder HiFi DNA Assembly Cloning kit (New England Biolabs). Standard transformation procedures and plasmid isolation for *Escherichia coli* were used. To create a C-terminal mCherry fusion of histone H2B under the control of the *artA* promoter, the corresponding fragment of ~1.5 kb including the promoter sequences was amplified by PCR, using *A. flagrans* genomic DNA as template. The backbone of the plasmid containing H2B and mCherry was amplified and assembled with the promoter^11^.

Gene deletions were obtained by homologous recombination. Around 1 kb flanks homologous to the 5’ and 3’ regions of the targeted gene were amplified by PCR using *A. flagrans* genomic DNA as template. Both fragments containing 25 bp overlapping regions to the neighbouring fragment were assembled, with a hygromycin-B or geneticin resistance cassette in between, into the pJET1.2 vector (Thermo Fisher, digested with *Eco*RV) using the NEBuilder HiFi DNA Assembly Cloning kit.

For chimaeric protein recomplementation experiments, the region encoding the first four TM helices in GPCRs was amplified from cDNA of *C. elegans* and the region encoding the last three TM helices of GprC was amplified from genomic DNA of *A. flagrans*. The two half fragments were assembled under the control of the native promoter of *gprC* and introduced into the deletion strain of *gprC*. The full length of the GPCR genes was amplified from cDNA of *C. elegans* and ligated with the backbone containing 1.5 kb fragments upstream and downstream of the *gprC* open reading frame (ORF). TM helices were predicted by the server Phyre2 (http://www.sbg.bio.ic.ac.uk/~phyre2/html/page.cgi?id=index) and TMHMM 2.0 (https://services.healthtech.dtu.dk/services/TMHMM-2.0/).

For site-directed mutagenesis, mutated *gprC* genes with 1.5 kb left and right borders were assembled into the pJET1.2 vector containing the G418 cassette^12^. The *gprC* expression cassette was amplified from two fragments: (1) the first half fragments containing 1.5 kb left border and the sequences from the start codon until the mutated spot (the reverse primer contains the mutated gene sequence) and (2) the other half fragments containing 1.5 kb right border and the sequences from the mutated spot until the stop codon (the forward primer contains the mutated gene sequence). All amino acids were mutated into alanine using codon GCT.

For the C-terminal GFP fusion of GprC, the *gprC* ORF region excluding the stop codon or 120 bp of *citA* gene was amplified from gDNA of *A. flagrans* and *A. nidulans* individually. The backbone containing GFP and the hygromycin-B cassette was amplified from PNH21. The genes were expressed under the control of the constitutive *A. nidulans oliC* promoter. To express the *gprC* or *gasA* gene natively, the 1.5 kb fragments upstream of the gene ORFs were used as promoters.

For the bifluorescent complementation experiment, the GFP gene was split into N-terminal and C-terminal fragments as in *Botrytis cinerea*, and linkers were used between GFP fragments and the *A. flagrans* genes^44^. The C-terminal half of the GFP-encoding DNA fragment was fused at the 3′ end of *gprC* and the N-terminal half of GFP-encoding fragment, at the 5′ end of the *gasA* gene. Both constructs were under their native promoters. The *gprC-GFPC* and the *GFPN-gasA* fragments were ligated with the backbones containing G418 and hygromycin-B resistance cassettes, respectively. All plasmids are listed in Supplementary Table 2.

### RNA extraction, RT–qPCR and cDNA synthesis

To induce traps for RNA extraction, 10^6^ *A. flagrans* spores were incubated on LNA covered with a cellophane membrane for 24 h at 28 °C. Individuals (10^4^) of a mixed *C. elegans* population were added to the membrane and co-incubated at 28 °C for 24 h to induce trap formation. The uninduced group was treated with the same volume of double-distilled H_2_O. Afterwards, mycelia were collected from cellophane membranes on LNA and ground in liquid N_2_. Total RNA was extracted with Trizol reagent (Invitrogen). DNase digestion was performed using the Turbo DNA-free kit (Invitrogen) and RNA was diluted to 50 ng μl^−1^. The SensiFast SYBR and fluorescein One Step kit (Bioline) was used for the RT–qPCR analysis on a CFX Connect Real-Time PCR Detection System (Bio-Rad). Each reaction mixture contained 0.2 μM oligonucleotides (Supplementary Table 3; Eurofins Genomics Europe) and 100 ng of RNA in a 20 μl total volume. Melting curve analysis was performed to assess the specific amplification of DNA. Fold changes were calculated using the formula 2^−(ΔΔCt)^, with ΔΔCt being ΔCt (treatment)−ΔCt (control), ΔCt is Ct (target gene)−Ct (actin) and Ct is the threshold cycle. The gamma actin orthologue DFL_002353 was used as internal reference gene for normalization. RT–qPCR was performed with three biological replicates. The First-Strand cDNA Synthesis kit (Thermo Fisher) was used for cDNA synthesis and RT–PCR.

### Fractionation of mitochondria and plasma membrane

*A. flagrans* protoplast was applied to the Yeast Mitochondria Isolation Kit (Sigma-Aldrich) and mitochondria were isolated as described in the manufacturer’s protocol using detergent lysis. In the first centrifugation step of 400*g*, mitochondria were obtained in the supernatant (S1) and in the pellet (P1). We used the supernatant for the second centrifugation of 11,000*g*, where mitochondria sedimented in the pellet (P2). The second supernatant (S2) was used for the third centrifugation at 100,000*g*, where the plasma membrane was sedimented in the pellet (P3). All pellet samples were used for digestion by proteinase K. Proteins were analysed in a western blot using anti-GFP antibody (11814460001, Roche) and anti-mouse IgG (Fab specific)-peroxidase antibody (A2304, Sigma-Aldrich). The protein amount was normalized to the volume in the original protein extract. Before blocking, the nitrocellulose membrane was stained using Ponceau S (0.1% Ponceau red dye, 5% glacial acetic acid) for 15 min.

### Yeast two-hybrid assay

This work followed the user manual of the Matchmaker Gold yeast two-hybrid system (Clontech). Genes of *gprC, gasA* and the 111 amino acid C-terminal tail of *gprC* were amplified from the cDNA of *A. flagrans*. The PCR products were ligated into pGBKT7 and pGADT7 vectors. In the constructs, BD and AD domains were fused at the N terminus of GasA and the C terminus of GprC fragments individually. Yeast strains AH109 and Y187 were used for transformation of BD and AD vectors. The two strains were mated for interaction detection. Dilution series of strains were grown on SD-LW (leucine^−^, tryptophan^−^) and SD-LWH (leucine^−^, tryptophan^−^ and histidine^−^) agar plates for 3–5 days.

### Measurement of oxygen consumption rate

The analysis was performed with an XF24 extracellular flux analyser (Seahorse XFe24)^45^. Spores (~10^3^) of the *A. flagrans* WT and mutant strains suspended in 10 μl liquid low-nutrient medium were incubated in XF24 Islet capture microplates for 24 h at 28 °C. Then, ~50 nematode adults were added in wells for induction of 6 h, while the same volume of sterile water was used as a control (uninduced). After that, free living worms were washed off carefully. Then, the wells were filled with 200 μl liquid low-nutrient medium for OCR detection. Wells with 210 μl liquid low-nutrient medium were used as control.

### Application of 8’-bromo-cAMP

8’-bromo-cAMP (Sigma) was used as the analogue of cAMP, with 1 mg dissolved into 20 μl 1 M ammonia as stock solution of 50 mg ml^−1^ (122.52 mM). The working concentration was 5 mM suspended in the melted LNA medium, which was cooled down to the proper temperature.

### Protein extraction and immunoblotting

Around 10^6^ spores of *A. flagrans* were inoculated on cellophane on LNA plates and incubated for 5 days at 28 °C. For induction, 10,000 N2 nematodes were applied on the grown hyphae for co-incubation of 3 h at 28 °C. Afterwards, worms were washed off with double-distilled H_2_O. The mycelia were collected and immediately frozen in liquid nitrogen for protein extraction. Mycelia from four LNA plates were collected into Eppendorf tubes and ground in liquid nitrogen. Protein extraction buffer (500 μl; 20 mM Tris-HCl, pH 8.0, 0.05% Triton-X-100, 150 mM NaCl) containing 1 mM PMSF was added into each tube and incubated on ice for 20 min. The samples were centrifuged at 17,000*g* at 4 °C. The supernatants were collected. The protein concentration was measured using the Bradford protein assay and all the samples were adjusted to the same concentration with protein extraction buffer. Samples with 5× SDS loading buffer and 10 mM dithiothreitol were denatured at 95 °C for 10 min. Then, denatured samples were loaded onto a 10% SDS polyacrylamide gel and blotted to a nitrocellulose membrane. For immunodetection, anti-phospho-p38 MAP kinase (Tyr180/Tyr182) antibodies (9211, Cell Signaling Technology; dilution 1:1,000) against phosphorylated HogA, anti-phospho-p44/42 MAPK (Erk1/2) (Thr202/Tyr204) antibodies (9101, Cell Signaling Technology; dilution 1:1,000) against phosphorylated MakA/MakB, anti-Histone H3 (DFL_003537) (ab1791, abcam; dilution 1:2,000) antibodies, and anti-rabbit IgG (whole molecular)-peroxidase antibody (A0545, Sigma-Aldrich; dilution 1:10,000) and anti-mouse IgG (Fab specific)-peroxidase antibody (A2304, Sigma-Aldrich; dilution 1:10,000) were used.

### Microscopy

To induce trap formation for microscopy, ~10^4^ spores of *A. flagrans* were inoculated on thin LNA on top of microscopic slides and ~200 individuals of *C. elegans* were added. The compound Ascr#18 (MedChenExpress) was used for induction. Co-incubation was performed at 28 °C in darkness for 20 h. To visualize ROS, we used the CellROX orange reagent (Invitrogen).

Conventional fluorescence images were captured at room temperature using a Zeiss Plan-Apochromat ×63/1.4 Oil DIC, EC Plan-Neofluar ×40/0.75, EC Plan-Neofluar ×20/0.50 or EC Plan-Neofluar ×10/0.30 objective attached to a Zeiss AxioImager Z.1 and AxioCamMR. Images were collected using ZEN 2012 Blue Edition. The Zeiss LSM 900 with Airyscan2 was used for confocal microscopy. Colour images were acquired using the AxioCam 105 colour. Confocal images were captured at room temperature using a Leica HCX PL APO ×63/1.4 oil objective attached to a Leica TC SP5 and conventional photomultiplier tube detectors (Leica). Images were collected using the AxioVision software. Cell fluorescence was measured using ImageJ. (https://theolb.readthedocs.io/en/latest/imaging/measuring-cell-fluorescence-using-imagej.html). CTCF (corrected total cell fluorescence) = integrated density − (area of selected cell × mean fluorescence of background readings).

### Modelling and docking

Models for GprC, SRBC64 and DAF37 are available in the AlphaFold Protein Structure Database^46^. They showed high pLDDT scores (>0.85) for the TM helices, which in principle allows docking studies^47^. Mutant receptors and chimaeric proteins were modelled using the AF2 web server with five models per output, 24 max_recycles and 2 num_ensemble.

For protein–ligand docking with Autodock Vina, the transmembrane portions of the predicted GPCR structures were prepared using the ‘prepare_receptor’ tool of the ADFR suite^48^. Ligands were constructed using Avogadro^49^ and geometry optimized using Orca at a B3LYP/def2-TZVP level of theory. Following the default settings of the ‘prepare_ligand’ tool in the ADFR suite, all single bonds that are not included in the ascarylose ring were set to be rotable, resulting in 7–11 DOFs for the four ascarosides. Atomic charges of the ligand atoms were determined using the Gasteiger charge model^50^. After processing the pdbqt files, docking was performed with a box size of 40 Å around the centre of mass of the receptor and an exhaustiveness of 200 and 0.1 Å spacing. Binding affinities and structures of the first five binding poses were used.

### Statistics and reproducibility

Group sizes are described in the figure legends. Unless specifically noted, each experiment was repeated three or more times independently. Data were collected from three biological repeats, unless otherwise noted. Data shown in graphs or plots represent mean ± s.d., as indicated in figure legends. Plotted data points are shown. Details are given in the above methods and in source data files. Data diagrams and statistical analyses were performed with GraphPad Prism 8.0 and IBM SPSS Statistics 19. For statistical analysis, a two-sided unpaired Student’s *t*-test was performed, with *P* < 0.05 considered significant.

### Reporting summary

Further information on research design is available in the Nature Portfolio Reporting Summary linked to this article.

### Supplementary information

Supplementary Information Supplementary Tables 1–3.

Reporting Summary

### Source data

Source Data Fig. 1 Statistical source data.

Source Data Fig. 2 Source data western blots.

Source Data Fig. 3 Source data PCR and Southern blots.

## Figures and Tables

**Extended Data Table 1:**
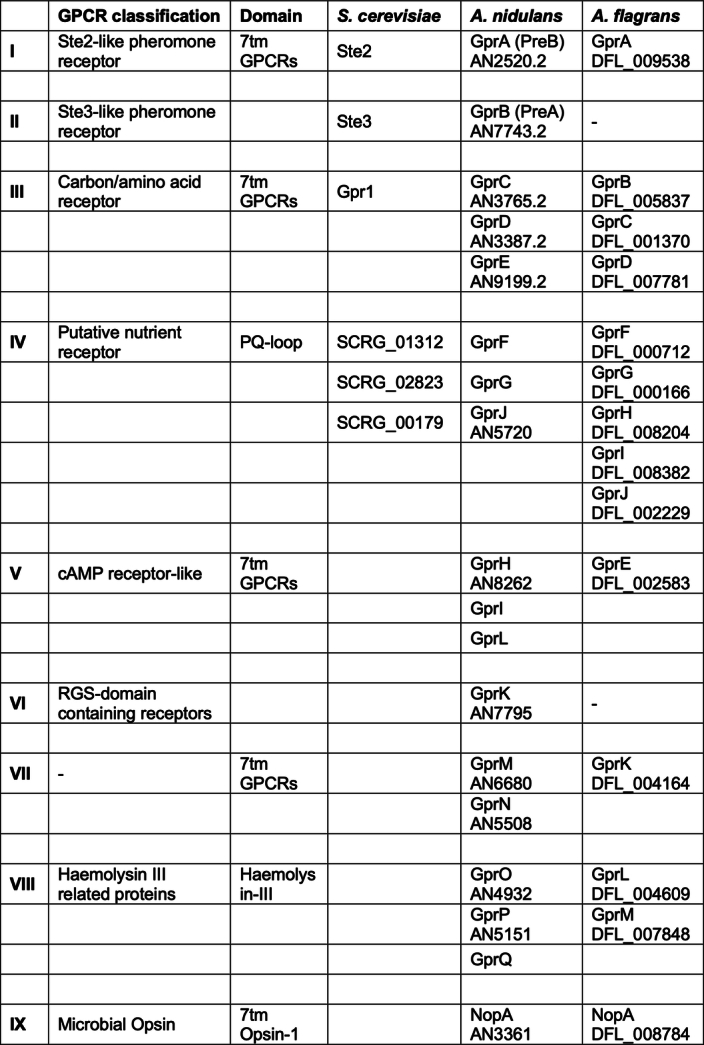
Identification of GPCRs in *A. flagrans* and comparison with GPCRs in other fungi

By standard protein Blast, AN2520 from *A. nidulans* and DFL_009538 from *A. flagrans* share 26.28% similarity and the e-value is 6e-19; Gpr1 of *S. cerevisiae* shares 29.43% and 21.53% similarities with DFL_005837 and DFL_007781 (e-values are 4e-39 and 0.039 respectively); and AN3387 and DFL_001370 share similarity of 24.19% (e-value is 0.049); AN5720 shares similarities of 41.81%, 31.34% and 24.17% with DFL_008204, DFL_008382 and DFL_000166 (e-values are 6e-73, 2e-39 and 4e-16 individually); and DFL_008204 shares 33.33% similarity with DFL_002229 (e-value is 0.026); DFL_008382 shares 28.74% similarity with DFL_000712 (e-value is 0.001); AN8262 and DFL_002583 share 34.75% similarity (e-value is 1e-75); AN6690 and DFL_004164 share 38.05% similarity (e-value is 6e-98); AN4932 shares similarities of 31.77%, 52,9% and 38.55% with DFL_007848, DFL_004609 and DFL_007880 individually (e-values are 8e-27, 1e-110 and 4e-15); AN3361 and DFL_008784 share 34.81 similarity and e-value is 8e-51.

**Extended Data Table 2:**
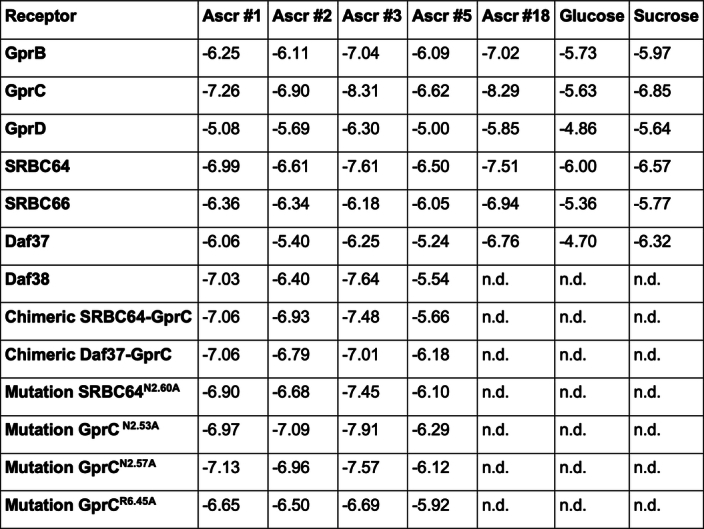
Binding affinities of the best binding poses in kcal/mol

**Extended Data Table 3:**
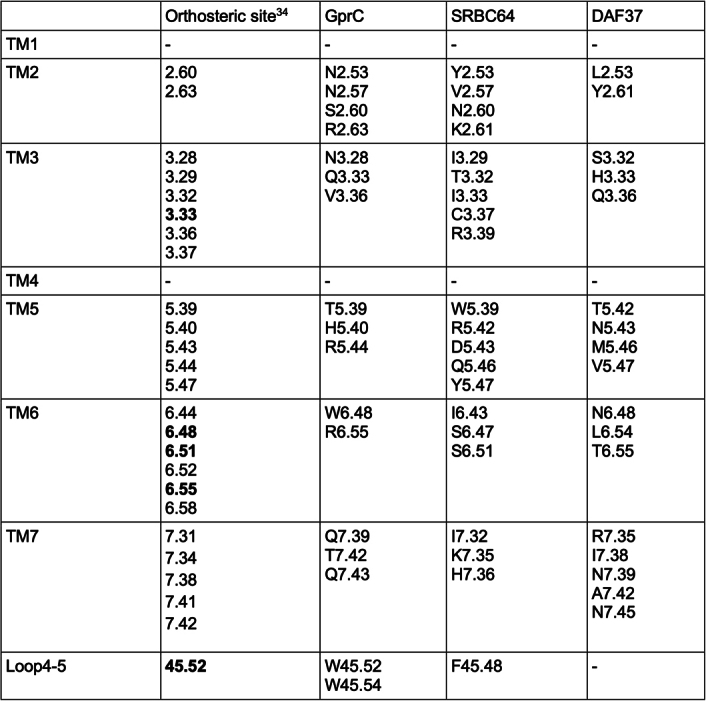
Coordinating residues of three receptors with ascarosides by the Ballesteros–Weinstein numbering

Bold numbers indicate 5 positions that show highest interaction frequencies of about 40% in >200 GPCR class A crystal structures from ref. [34]. The most conserved residue in TM7 could not be clearly defined for GprC and SRBC64, therefore, the most likely positions are shown.

**Extended Data Table 4:**
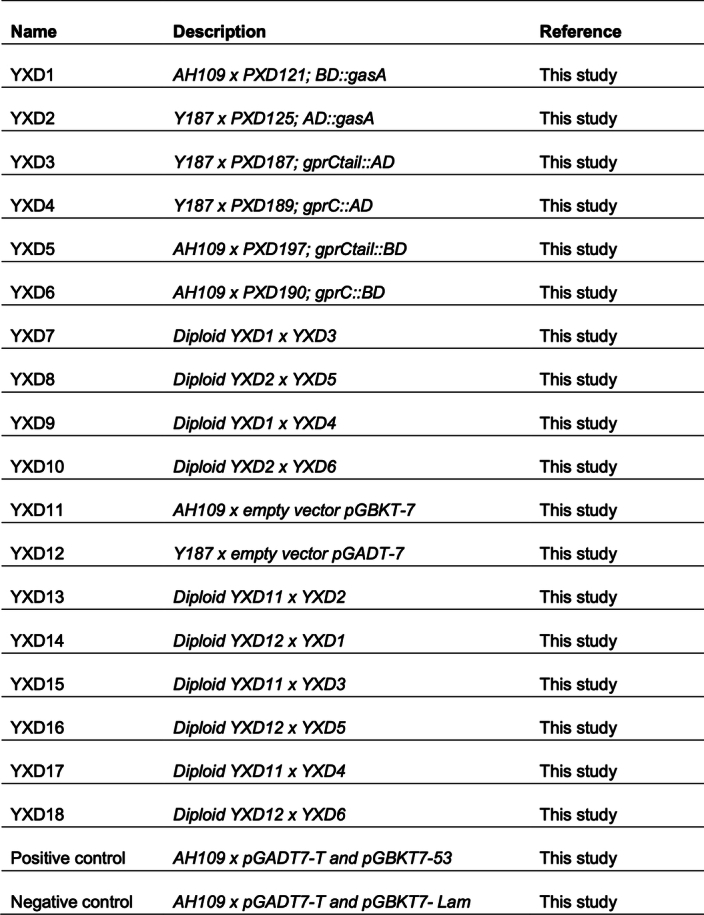
*S. cerevisiae* strains used in this study

## Data Availability

All data generated or analysed during this study are included in this published article or provided as source data files. The *Arthrobotrys flagrans* genome database used in this study is available at the National Center for Biotechnology Information GenBank under the accession number PRJNA494930. References to this accession number can be found throughout this paper. Source data are provided with this paper.

## References

[1] Lefkowitz RJ (2013). A brief history of G-protein coupled receptors (Nobel lecture). Angew. Chem. Int. Ed. Engl..

[2] Yang D (2021). G protein-coupled receptors: structure- and function-based drug discovery. Signal Transduct. Target. Ther..

[3] Weis WI, Kobilka BK (2018). The molecular basis of G protein-coupled receptor activation. Annu. Rev. Biochem..

[4] Benard G (2012). Mitochondrial CB_1_ receptors regulate neuronal energy metabolism. Nat. Neurosci..

[5] Versele M, Lemaire K, Thevelein JM (2001). Sex and sugar in yeast: two distinct GPCR systems. EMBO Rep..

[6] Kou Y, Tan YH, Ramanujam R, Naqvi NI (2017). Structure–function analyses of the Pth11 receptor reveal an important role for CFEM motif and redox regulation in rice blast. New Phytol..

[7] Hsueh YP, Mahanti P, Schroeder FC, Sternberg PW (2013). Nematode-trapping fungi eavesdrop on nematode pheromones. Curr. Biol..

[8] Yu X (2021). Fatal attraction of *Caenorhabditis elegans* to predatory fungi through 6-methyl-salicylic acid. Nat. Commun..

[9] Butcher RA (2017). Small-molecule pheromones and hormones controlling nematode development. Nat. Chem. Biol..

[10] Fischer R, Requena N (2022). Small-secreted proteins as virulence factors in nematode-trapping fungi. Trends Microbiol..

[11] Youssar L (2019). Intercellular communication is required for trap formation in the nematode-trapping fungus *Duddingtonia flagrans*. PLoS Genet..

[12] Wernet N, Wernet V, Fischer R (2021). The small-secreted cysteine-rich protein CyrA is a virulence factor of *Duddingtonia flagrans* during the *Caenorhabditis elegans* attack. PLoS Pathog..

[13] Lin HC (2023). Key processes required for the different stages of fungal carnivory by a nematode-trapping fungus. PLoS Biol..

[14] Kim K (2009). Two chemoreceptors mediate developmental effects of dauer pheromone in *C. elegans*. Science.

[15] Park JY, Joo HJ, Park S, Paik YK (2019). Ascaroside pheromones: chemical biology and pleiotropic neuronal functions. Int. J. Mol. Sci..

[16] Zhen Z (2018). MAP kinase Slt2 orthologs play similar roles in conidiation, trap formation, and pathogenicity in two nematode-trapping fungi. Fungal Genet. Biol..

[17] Kuo CY, Chen SA, Hsueh YP (2020). The high osmolarity glycerol (hog) pathway functions in osmosensing, trap morphogenesis and conidiation of the nematode-trapping fungus *Arthrobotrys oligospora*. J. Fungi.

[18] Chen SA, Lin HC, Schroeder FC, Hsueh YP (2021). Prey sensing and response in a nematode-trapping fungus is governed by the MAPK pheromone response pathway. Genetics.

[19] Chen SA, Lin HC, Hsueh YP (2022). The cAMP-PKA pathway regulates prey sensing and trap morphogenesis in the nematode-trapping fungus *Arthrobotrys oligospora*. G3.

[20] Suelmann R, Fischer R (2000). Mitochondrial movement and morphology depend on an intact actin cytoskeleton in *Aspergillus nidulans*. Cell Motil. Cytoskel..

[21] Streng C (2021). Fungal phytochrome chromophore biosynthesis at mitochondria. EMBO J..

[22] Hsueh YP (2017). Nematophagous fungus *Arthrobotrys oligospora* mimics olfactory cues of sex and food to lure its nematode prey. eLife.

[23] Wang BL (2018). Integrated metabolomics and morphogenesis reveal volatile signaling of the nematode-trapping fungus *Arthrobotrys oligospora*. Appl. Environ. Microbiol..

[24] Zhang HX (2012). Morphology regulatory metabolites from *Arthrobotrys oligospora*. J. Nat. Prod..

[25] Huang J, Zheng X, Tian M, Zhang K (2023). Ammonia and nematode ascaroside are synergistic in trap formation in *Arthrobotrys oligospora*. Pathogens.

[26] Wernet V, Wäckerle J, Fischer R (2022). The STRIPAK component SipC is involved in morphology and cell-fate determination in the nematode-trapping fungus *Duddingtonia flagrans*. Genetics.

[27] Pfanner N, Warscheid B, Wiedemann N (2019). Mitochondrial proteins: from biogenesis to functional networks. Nat. Rev. Mol. Cell Biol..

[28] Wozny MR (2023). In situ architecture of the ER–mitochondria encounter structure. Nature.

[29] Yang Y, Yang E, An Z, Liu X (2007). Evolution of nematode-trapping cells of predatory fungi of the Orbiliaceae based on evidence from rRNA-encoding DNA and multiprotein sequences. Proc. Natl Acad. Sci. USA.

[30] Sun J, Singh V, Kajino-Sakamoto R, Aballay A (2011). Neuronal GPCR controls innate immunity by regulating noncanonical unfolded protein response genes. Science.

[31] Eberhardt J, Santos-Martins D, Tillack AF, Forli S (2021). AutoDock Vina 1.2.0: new docking methods, expanded force field, and Python bindings. J. Chem. Inf. Model..

[32] Trott O, Olson AJ (2010). AutoDock Vina: improving the speed and accuracy of docking with a new scoring function, efficient optimization, and multithreading. J. Comput. Chem..

[33] Lemaire K, Van de Velde S, Van Dijck P, Thevelein JM (2004). Glucose and sucrose act as agonist and mannose as antagonist ligands of the G protein-coupled receptor Gpr1 in the yeast *Saccharomyces cerevisiae*. Mol. Cell.

[34] Chan HCS, Li Y, Dahoun T, Vogel H, Yuan S (2019). New binding sites, new opportunities for GPCR drug discovery. Trends Biochem. Sci..

[35] Wheatley M (2007). Extracellular loops and ligand binding to a subfamily of family A G-protein-coupled receptors. Biochem. Soc. Trans..

[36] Ragnarsson L, Andersson A, Thomas WG, Lewis RJ (2015). Extracellular surface residues of the alpha1B-adrenoceptor critical for G protein-coupled receptor function. Mol. Pharmacol..

[37] Vidal B (2018). An atlas of *Caenorhabditis elegans* chemoreceptor expression. PLoS Biol..

[38] Isberg V (2014). GPCRDB: an information system for G protein-coupled receptors. Nucleic Acids Res..

[39] Ballesteros JA, Weinstein H (1995). Integrated methods for the construction of three-dimensional models and computational probing of structure–function relations in G protein-coupled receptors. Methods Neurosci..

[40] Irannejad R (2013). Conformational biosensors reveal GPCR signalling from endosomes. Nature.

[41] Hebert-Chatelain E (2016). A cannabinoid link between mitochondria and memory. Nature.

[42] Mohammad Nezhady MA, Rivera JC, Chemtob S (2020). Location bias as emerging paradigm in GPCR biology and drug discovery. iScience.

[43] Mendizabal-Zubiaga J (2016). Cannabinoid CB(1) receptors are localized in striated muscle mitochondria and regulate mitochondrial respiration. Front. Physiol..

[44] Schumacher J (2012). Tools for *Botrytis cinerea*: new expression vectors make the gray mold fungus more accessible to cell biology approaches. Fungal Genet Biol..

[45] Bonnighausen J (2015). Disruption of the GABA shunt affects mitochondrial respiration and virulence in the cereal pathogen *Fusarium graminearum*. Mol. Microbiol..

[46] Varadi M (2022). AlphaFold Protein Structure Database: massively expanding the structural coverage of protein-sequence space with high-accuracy models. Nucleic Acids Res..

[47] Lee S (2023). Evaluating GPCR modeling and docking strategies in the era of deep learning-based protein structure prediction. Comput. Struct. Biotechnol. J..

[48] Ravindranath PA, Forli S, Goodsell DS, Olson AJ, Sanner MF (2015). AutoDockFR: advances in protein-ligand docking with explicitly specified binding site flexibility. PLoS Comput. Biol..

[49] Hanwell MD (2012). Avogadro: an advanced semantic chemical editor, visualization, and analysis platform. J. Cheminform..

[50] Gasteiger J, Marsili M (1980). Iterative partial equalization of orbital electronegativity—a rapid access to atomic charges. Tetrahedron.

