# Supplementary material for: GprC of the nematode-trapping fungus Arthrobotrys flagrans activates mitochondria and reprograms fungal cells for nematode hunting
Source: Nat Microbiol. 2024 Jun 14;9(7):1752–63. doi: 10.1038/s41564-024-01731-9 (PMC11222155; doi:10.1038/s41564-024-01731-9)
Supplement: Supplementary file 1 — Supplementary Tables 1–3. [file 41564_2024_1731_MOESM1_ESM.pdf]

nature microbiology

Supplementary information

https://doi.org/10.1038/s41564-024-01731-9

GprC of the nematode-trapping fungus *Arthrobotrys flagrans* activates mitochondria and reprograms fungal cells for nematode hunting

In the format provided by the authors and unedited

Supplementary Table 1: *A. flagrans* strains used in this study.

Strain	Genotype	Reference
SXD39	*ΔgasA PXD66; hph*	This study
SXD40	*ΔgasB PXD64; hph*	This study
SXD42	*SXD39 x gasA(p)::gasA::gasA(t) PXD71; G418*	This study
SXD43	*SXD40 x gasB(p)::gasB::gasB(t) PXD72; G418*	This study
SXD44	*SXD39 x artA(p)::h2b:mCherry::tuB(t) PXD73; G418*	This study
SXD57	*ΔgprC PXD103; hph*	This study
SXD62	*SXD57 x gprC(p)::gprC::gprC(t) PXD106; G418*	This study
SXD82	*SXD57 x gprC(p)::daf-37(N)::gprC(C)::gprC(t) PXD128; G418*	This study
SXD83	*SXD57 x gprC(p)::srbc-64(N)::gprC(C)::gprC(t)PXD127; G418*	This study
SXD84	*SXD57 x gprC(p)::daf-38(N)::gprC(C)::gprC(t) PXD133; G418*	This study
SXD85	*SXD57 x gprC(p)::octr-1(N)::gprC(C)::gprC(t) PXD134; G418*	This study
SXD99	*SXD57 x gprC(p)::srbc-66(N)::gprC(C)::gprC(t)PXD132; G418*	This study
SXD116	*SXD57 x artA(p)::h2b:mCherry::tuB(t) PXD73; G418*	This study
SXD117	*oliC(p)::gprC::GFP::tub(t) PXD166; hph*	This study
SXD121	*gprC(p)::gprC::GFP::tub(t) PXD167; hph*	This study
SXD122	*gasA(p)::GFPN::gasA::tub(t) PXD157; hph*<br>*gprC(p)::gprC::GFPC::tub(t) PXD159; G418*	This study
SXD123	*SXD57 x gprC(p)::gprC*$^{S2.41A}$*::gprC(t) PXD142; G418*	This study
SXD124	*SXD57 x gprC(p)::gprC*$^{R6.45A}$*::gprC(t) PXD143; G418*	This study
SXD125	*SXD57 x gprC(p)::srbc-66::gprC(t) PXD151; G418*	This study
SXD126	*SXD57 x gprC(p)::gprC*$^{N2.53A}$*::gprC(t) PXD152; G418*	This study
SXD127	*SXD57 x gprC(p)::gprC*$^{N2.57A}$*::gprC(t) PXD162; G418*	This study
SXD128	*SXD57 x gprC(p)::srbc-64(N)*$^{N2.60A}$*::gprC(C)::gprC(t) PXD163; G418*	This study
SXD129	*SXD39 x gasA(p)::GFP::gasA::gasA(t) PXD170; G418*	This study

SXD135	*SXD57 x gprC(p)::gprC::GFP::gprC(t) PXD176; G418*	This study
SXD162	*oliC(p)::citA*^{N}*::GFP::tub(t) PXD209; hph*	This study
SMW01	*ΔgprA PMW01; hph*	This study
SMW02	*ΔgprB PMW02; hph*	This study
SMW04	*ΔgprD PMW04; hph*	This study
SMW05	*ΔgprE PMW05; hph*	This study
SMW06	*ΔgprF PMW06; hph*	This study

Supplementary Table 2: Plasmids used in this study.

Name	Description	Reference
PXD64	*ΔgasB; Amp; hph*	This study
PXD66	*ΔgasA; Amp; hph*	This study
PXD71	*gasA(p)::gasA::gasA(t); Amp; G418*	This study
PXD72	*gasB(p)::gasB::gasB(t); Amp; G418*	This study
PXD73	*artA(p)::h2b:mCherry::tuB(t); Amp*	This study
PXD103	*ΔgprC; Amp; hph*	This study
PXD106	*gprC(p)::gprC::gprC(t); Amp; G418*	This study
PXD121	*pGBKT-7::gasA; Kan*	This study
PXD125	*pGADT-7::gasA; Amp*	This study
PXD127	*gprC(p)::srbc-64(N)::gprC(C)::gprC(t); Amp; G418*	This study
PXD128	*gprC(p)::daf-37(N)::gprC(C)::gprC(t); Amp; G418*	This study
PXD132	*gprC(p)::srbc-66(N)::gprC(C)::gprC(t); Amp; G418*	This study
PXD133	*gprC(p)::daf-38(N)::gprC(C)::gprC(t); Amp; G418*	This study
PXD134	*gprC(p)::octr-1(N)::gprC(C)::gprC(t); Amp; G418*	This study
PXD142	*gprC(p)::*$gprC^{S2.41A}$*::gprC(t); Amp; G418*	This study
PXD143	*gprC(p)::*$gprC^{R6.45A}$*::gprC(t); Amp; G418*	This study
PXD151	*gprC(p)::srbc-66::gprC(t); Amp; G418*	This study
PXD152	*gprC(p)::*$gprC^{N2.53A}$*::gprC(t); Amp; G418*	This study
PXD157	*gasA(p)::GFPN::gasA::tub(t); Amp; hph*	This study
PXD159	*gprC(p)::gprC::GFPC::tub(t); Amp; G418*	This study
PXD162	*gprC(p)::*$gprC^{N2.57A}$*::gprC(t); Amp; G418*	This study
PXD163	*gprC(p)::srbc-64*$(N)^{N2.60A}$*::gprC(C)::gprC(t); Amp; G418*	This study
PXD166	*oliC(p)::gprC::GFP::tub(t); Amp; hph*	This study
PXD167	*gprC(p)::gprC::GFP::tub(t); Amp; hph*	This study
PXD170	*gasA(p)::GFP ::gasA::tub(t); Amp; G418*	This study

PXD176	*gprC(p)::gprC::GFP::tub(t); Amp; G418*	This study
PXD187	*pGADT-7::gprCtail; Amp*	This study
PXD189	*pGADT-7::gprC; Amp*	This study
PXD190	*pGBKT-7::gprC; Kan*	This study
PXD197	*pGBKT-7::gprCtail; Kan*	This study
PXD209	*oliC(p)::citA*^{N}*::GFP::tub(t); hph*	This study
pMW01	*ΔgprA; trpC(p)::hph::trpC(t); Amp; hph*	This study
pMW02	*ΔgprB; trpC(p)::hph::trpC(t); Amp; hph*	This study
pMW04	*ΔgprD; trpC(p)::hph::trpC(t); Amp; hph*	This study
pMW05	*ΔgprE; trpC(p)::hph::trpC(t); Amp; hph*	This study
pMW06	*ΔgprF; trpC(p)::hph::trpC(t); Amp; hph*	This study

Supplementary 3: Oligonucleotides used in this study.

Name	Sequence (from 5' to 3')	Description
GasALB-pjet-ol-for	GATGGCTCGAGTTTTTCAGCAAGATACCTTC GTCATCATCAGATCA	*gasA* deletion
GasALB-H-ol-rev	GTTGACCTCCACTAGCATTACACTTTTTGAC GGTGTTTCTTTAGAAAAA	*gasA* deletion
GasARB-H-ol-for	ATGCTCTTTCCCTAAACTCCCCCCAACGAGT TCCCGACCAATGA	*gasA* deletion
GasARB-pjet-ol-rev	ATTGTAGGAGATCTTCTAGAAAGATAATACA TGAACACACACCAGG	*gasA* deletion
H-GasALB-ol-for	TTTTTTCTAAAGAAACACCGTCAAAAAGTGT AATGCTAGTGGAGGT	*gasA* deletion
H-GasARB-ol-rev	TGTTGGTCATTGGTCGGGAACTCGTTGGGG GGAGTTTAGGGAAA	*gasA* deletion
GasAko_up_for	CCTGCAAGAGGGGAGCAAA	*gasA* deletion
GasAko_down_rev	CCTCGATTACAATTGTTTGGAG	*gasA* deletion
GasA_LB_for	ACCTTCGTCATCATCAGATCA	*gasA* deletion
GasA_LB_rev	AAAAATGCCAGCGGCAGAG	*gasA* deletion
GasA_RB_rev	AATACATGAACACACACCAGG	*gasA* deletion
GasA_ORF_for	ATGGGTTGCAGCATGTCTAC	*gasA* deletion
GasA_ORF_rev	TTATATCAGACCGCAGTTTCTG	*gasA* deletion
GasBLB_pjet_ol_for	GATGGCTCGAGTTTTTCAGCAAGATCACCA CTGCAGTAACCTCTA	*gasB* deletion
GasBLB-H-ol-rev	GTTGACCTCCACTAGCATTACACTTTTTGGC TGACTTGCAATCTC	*gasB* deletion
GasBRB-H-ol-for	ATGCTCTTTCCCTAAACTCCCCCCACCGACT AAAAATACCAACATCC	*gasB* deletion
GasBRB-pjet-ol-rev	ATTGTAGGAGATCTTCTAGAAAGATTCCGAG ATAGCGGTGTTCA	*gasB* deletion
H-GasBLB-ol-for	TCGGTGAGATTGCAAGTCAGCCAAAAAGTG TAATGCTAGTGGAGGT	*gasB* deletion
H-GasBRB-ol-rev	GTCGGATGTTGGTATTTTTAGTCGGTGGGG GGAGTTTAGGGAAA	*gasB* deletion
GasB_KO_up_for_n	TGACTAGTAGCCCAAATGAAC	*gasB* deletion
GasB_KO_down_rev_n	TAGACGGGCTGCATTTTGG	*gasB* deletion
GasB_LB_for	CACCACTGCAGTAACCTCTA	*gasB* deletion
GasB_RB_for	CCGACTAAAAATACCAACATCC	*gasB* deletion
GasB_RB_rev	TCCGAGATAGCGGTGTTCA	*gasB* deletion
GasB_ORF_for	ATGGGTGGCTGCATGTCG	*gasB* deletion
GasB_ORF_rev	TTATAAGATACCAGAGTCCTTGA	*gasB* deletion
BB_TrpC P_for	AAGTGTAATGCTAGTGGAGGT	Recomplement
BB_Pjet_rev	ATCTTGCTGAAAAACTCGAGC	Recomplement
GasALB_recomp_ol_for	GATGGCTCGAGTTTTTCAGCAAGATCGTAAC GACGCCGATTGAAT	Recomplement
GasARB_recomp_ol_rev	GTTGACCTCCACTAGCATTACACTTCCTCGA TTACAATTGTTTGGAG	Recomplement
GasBLB_recomp_ol_for	GATGGCTCGAGTTTTTCAGCAAGATGGAGG GGTTGTTAAGGATGT	Recomplement
GasBRB_recomp_ol_rev	GTTGACCTCCACTAGCATTACACTTTCCGAG ATAGCGGTGTTCA	Recomplement

gprCre_ol_for	GATGGCTCGAGTTTTTCAGCAAGATCCCAAA TCCATGGTGAACCA	Recomplement
gprCre_ol_rev	GTTGACCTCCACTAGCATTACACTTACTCCC TATTAGCTTTCATGG	Recomplement
artA(p)-h2b-fw	CTCTTTCCCTAAACTCCCCCCACCTCAACGA ACCGCTTACA	*artA* reporter assay
artA(p)-h2b-rev	CGGCGGCTTTTGGTGGCATGGCAGGCCAAA GAAGTCAAG	*artA* reporter assay
GprCLB_pjet_ol_for	GATGGCTCGAGTTTTTCAGCAAGATCCTTGT TTCGCCATGACATG	*gprC* deletion
GprCLB_H_ol_rev	GTTGACCTCCACTAGCATTACACTTGGTTGC GTCAGTGTATTATCA	*gprC* deletion
GprCRB_H_ol_for	ATGCTCTTTCCCTAAACTCCCCCCACCGCG AAGAGAGTAATGAAC	*gprC* deletion
GprCRB_pjet_ol_rev	ATTGTAGGAGATCTTCTAGAAAGATCACTGA TCCCCTGTATATTGA	*gprC* deletion
H_GprCLB_ol_for	CGGATGATAATACACTGACGCAACCAAGTG TAATGCTAGTGGAGGT	*gprC* deletion
H_GprCRB_ol_rev	TTCTAGTTCATTACTCTCTTCGCGGTGGGGG GAGTTTAGGGAAA	*gprC* deletion
GprCLB_for	CCTTGTTTCGCCATGACATG	*gprC* deletion
GprCRB_rev	CACTGATCCCCTGTATATTGA	*gprC* deletion
GprCORF_for	ATGGCCTTCACGACACTTTC	*gprC* deletion
GprCORFin_rev	TGTTTCCGAAATCCAGACTTC	*gprC* deletion
GprCORF_rev	TTACTCTCTTCGCGGTCCC	*gprC* deletion
GprCko_up_for	AAAAGCAAATGGAATGGACCC	*gprC* deletion
GprCko_down_rev	ACTCCCTATTAGCTTTCATGG	*gprC* deletion
SRBC64_3rLB_ol_for	CGGATGATAATACACTGACGCAACCATGCC TGAAATAGTAATAATCTTG	Chimeric protein
SRBC64N_3rC_ol_rev	CAGCCATGTGTCATCACATATCGAATTGAAT TTGGTCTCTTGAGAC	Chimeric protein
gprC_C_for	TTCGATATGTGATGACACATGG	Chimeric protein
gprC_LB_rev	GGTTGCGTCAGTGTATTATCA	Chimeric protein
Daf37_3rLB_ol_for-n	CGGATGATAATACACTGACGCAACCATGGA TGTCATTGGGAACAT	Chimeric protein
Daf37N_3rC_ol_rev-n	CAGCCATGTGTCATCACATATCGAAAGAATA TACCTGCTGATAAATAG	Chimeric protein
SRBC66_3rLB_ol_for	CGGATGATAATACACTGACGCAACCATGTC AGCCATTACTATAACTTG	Chimeric protein
SRBC66N_3rC_ol_rev	CAGCCATGTGTCATCACATATCGAATTGAAC CTGGTCTCGTAACG	Chimeric protein
SRBC66C_3rRB_ol_rev	CATTAATTATCAACTTCTAGTTCATTAATATG TAGTAATAACTGTTGAAC	Chimeric protein
octr_3rLB_ol_for	CGGATGATAATACACTGACGCAACCATGTG GAACCTTAACTGCAGT	Chimeric protein
octrN_3rC_ol_rev	CAGCCATGTGTCATCACATATCGAAATCAGT GCTAACGGAGCACT	Chimeric protein
Daf38_3rLB_ol_for	CGGATGATAATACACTGACGCAACCATGCTT CTCCCTTCAAACTTG	Chimeric protein
Daf38N_3rC_ol_rev	CAGCCATGTGTCATCACATATCGAACCAGAA CTCGGCGTCAAA	Chimeric protein

GprC_Asc1_Polic_for	TCACAATCGATCCAACCGGCGCGCCATGGC CTTCACGACACTTTC	GprC-GFP fusion
GprC_GFP_PacI_ol_rev	TCACCCTTGGAAACCATCTTAATTAACTCTC TTCGCGGTCCCG	GprC-GFP fusion
BB_pacI_GFP_for	TTAATTAAGATGGTTTCCAAGGG	GprC-GFP fusion
BB_Polic_AscI_rev	GGCGCGCCGGTTGGAT	GprC-GFP fusion
GprCLB_ol_for	TTTCCCTAAACTCCCCCCACTGCAGCCCAAA TCCATGGTGAACCA	GprC-GFP fusion
GprC_GFP_PacI_ol_rev	TCACCCTTGGAAACCATCTTAATTAACTCTC TTCGCGGTCCCG	GprC-GFP fusion
GasALB_hph_ol_for	TTTCCCTAAACTCCCCCCACTGCAGCGTAAC GACGCCGATTGAAT	GasA-GFP fusion
GasA_pac1GFP_ol_rev	CACCCTTGGAAACCATCTTAATTAATATCAG ACCGCAGTTTCTGA	GasA-GFP fusion
citA40AA_Asc1_ol_for	TCACAATCGATCCAACCGGCGCGCCATGGC TTCTACACTCAGACTT	CitA-GFP fusion
citA40AA_GFP_ol_rev	CACCCTTGGAAACCATCTTAATTAACTTGGT CTTGCCGGTAGAG	CitA-GFP fusion
BB_Tgluc_for	CGTATGTAGATAAGATGTATGATT	Split-GFP
GFPN_rev	GGCCATGATATAAACGTTGTG	Split-GFP
GasA_15aa_NgfpN_ol_fo r	CAGTCACAACGTTTATATCATGGCCAGATCC ACCTCCGCCTGAACCGCCTCCACCAGATGG GCCTCCACCATGGGTTGCAGCATGTCTAC	Split-GFP
GasA_Tgluc_ol_rev	TAATCATACATCTTATCTACATACGTTATATC AGACCGCAGTTTCTG	Split-GFP
GasB_15aa_NgfpN_ol_fo r	CAGTCACAACGTTTATATCATGGCCAGATCC ACCTCCGCCTGAACCGCCTCCACCAGATGG GCCTCCACCATGGGTGGCTGCATGTCG	Split-GFP
GasB_Tgluc_ol_rev	TAATCATACATCTTATCTACATACGTTATAAG ATACCAGAGTCCTTGA	Split-GFP
GFPC_for	GACAAGCAAAAAAATGGAATCAAG	Split-GFP
GprC_AscI_Polic_for	TCACAATCGATCCAACCggcgcgccATGGCCTT CACGACACTTTC	Split-GFP
GprC_17aa_CgfpC_rev	CCTTGATTCCATTTTTTTGCTTGTCGTGGTT CATGACCTTCTGTTTCAGGTCGTTCGGGATC TTGCAGGCCGGGCGGCCTCTCTTCGCGGT CCCG	Split-GFP
3rre_ol_for	GATGGCTCGAGTTTTTCAGCAAGATCCCAAA TCCATGGTGAACCA	Site-mutation
3rre-R6.45A -rev	GGATGAAAGCAAAAGCCCAG	Site-mutation
3rre-R6.45A-for	CTGGGCTTTTGCTTTCATCC	Site-mutation
3rre_ol_rev	GTTGACCTCCACTAGCATTACACTTACTCCC TATTAGCTTTCATGG	Site-mutation
3rre-S2.41A-rev	CAAAATCAAAGCATGTCGAAAATG	Site-mutation
3rre-S2.41A-for	CATTTTCGACATGCTTTGATTTTG	Site-mutation
3rre-N2.57A-rev	GCTCTAATGCTTCCACATCC	Site-mutation
3rre-N2.57A-for	GGATGTGGAAGCATTAGAGC	Site-mutation
3rre-N2.53A-rev	GCTAGCTATGAAATCTGTAGG	Site-mutation
3rre-N2.53A-for	CCTACAGATTTCATAGCTAGC	Site-mutation
SRBC64-N2.60A-rev	TTCCCCATGCTAAGATCTACT	Site-mutation
SRBC64-N2.60A-for	AGTAGATCTTAGCATGGGGAA	Site-mutation

gprA_LB_for	GCTCGAGTTTTTCAGCAAGAT GCAGCTATAGTTGGTGGGAA	gprA deletion
gprA_LB_rev	ACCTCCACTAGCATTACACTT GTAATGTTTCCGCCATCTGG	gprA deletion
gprA_hph_for	AGATGGCGGAAACATTAC AAGTGTAATGCTAGTGGA	gprA deletion
gprA_hph_rev	CCTCTTGGGTTTTTGGGTA TGGGGGGAGTTTAGGGAAAG	gprA deletion
gprA_RB_for	CTTTCCCTAAACTCCCCCA TACCCAAAAACCCAAGAGGC	gprA deletion
gprA_RB_rev	GTAGGAGATCTTCTAGAAAGAT ACCGTAGAAGAAGGCCTTGA	gprA deletion
gprA_orf_for	ATGGACCAGATCGACTCGTT	Analytic PCR
gprA_orf_rev	TTAGAGGCCGTGTCCCGC	Analytic PCR
gprA_ex_for	CGCAAGTATGGATGCAACCT	Analytic PCR
gprA_ex_rev	TCTCTCAGCATCTAGCAGAG	Analytic PCR
gprB_LB_for	TTCCGGATGGCTCGAGTTTTTCAGCAAGATT TGACTCCTCGACGCCACA	gprB deletion
gprB_LB_rev	GATGTGTTGACCTCCACTAGCATTACACTTT TCATTTGGGAGGGGGACA	gprB deletion
gprB_hph_for	TTCATTTGGGAGGGGGACA AAGTGTAATGCTAGTGGAGG	gprB deletion
gprB_hph_rev	GAAATGCAATTCTAAGGAACGG TGGGGGGAGTTTAGGGAAAG	gprB deletion
gprB_RB_for	TGCTCTTTCCCTAAACTCCCCCA CCGTTCCTTAGAATTGCATTTC	gprB deletion
gprB_RB_rev	ATTGTAGGAGATCTTCTAGAAAGAT CGAATTAAACGTGGGCATATTAT	gprB deletion
gprB_orf_for	ATGGGCGACATGCCAGTTGA	Analytic PCR
gprB_orf_rev	CTATCTCCTCCTCGCACTC	Analytic PCR
gprB_ex_for	CAATCCAAAAGCTTCAGTGGA	Analytic PCR
gprB_ex_rev	CTCGGTGCATATGGATAGGT	Analytic PCR
gprD_LB_for	GATGGCTCGAGTTTTTCAGCAAGATATAGCT TTAGTACCCCACTTTG	gprD deletion
gprD_LB_rev	TGTTGACCTCCACTAGCATTACACTTCTCCG GCTAAATGTCACCA	gprD deletion
gprD_hph_for	GCTGGTGACATTTAGCCGGAG AAGTGTAATGCTAGTGGAGG	gprD deletion
gprD_hph_rev	TAAAGATGTGGCAAAATTGGAGATA TGGGGGGAGTTTAGGGAAA	gprD deletion
gprD_RB_for	TTTCCCTAAACTCCCCCA TATCTCCAATTTTGCCACATC	gprD deletion
gprD_RB_rev	AGGAGATCTTCTAGAAAGAT CAGGCATGTATGTCTGTACG	gprD deletion
gprD_orf_for	ATGACGCAACTCCCGTACTT	Analytic PCR

gprD_orf_rev	TCATCCAATGATCCGGCCGCC	Analytic PCR
gprD_ex_for	ACCCACTACCCCGAGGAAAT	Analytic PCR
gprD_ex_rev	CGCTCCGACAATATCTATGC	Analytic PCR
gprE_LB_for	GATGGCTCGAGTTTTTCAGCAAGAT CATGGTATTTCTCTTGACGTG	gprE deletion
gprE_LB_rev	GTTGACCTCCACTAGCATTACACTT GGTACGTTCTGCTGGTTTAC	gprE deletion
gprE_hph_for	GTAAACCAGCAGAACGTACC AAGTGTAATGCTAGTGGAGG	gprE deletion
gprE_hph_rev	CGGGAACTAGAACTGGGCAAAC TGGGGGGAGTTTAGGGAAA	gprE deletion
gprE_RB_for	CTTTCCCTAAACTCCCCCCA GTTTGCCCAGTTCTAGTTCC	gprE deletion
gprE_RB_rev	AGGAGATCTTCTAGAAAGAT TCTGAACGTTTTTGTCTCCC	gprE deletion
gprE_orf_for	ATGCCTCGCCAGTTAACAC	Analytic PCR
gprE_orf_rev	CTATTGGCTTCCGATCTTGATT	Analytic PCR
gprE_ex_for	TGCAAGGGTGCTACAGGTA	Analytic PCR
gprE_ex_rev	GCAGGGTCGAGATAGTGTC	Analytic PCR
gprF_LB_for	GCTCGAGTTTTTCAGCAAGATATCGTGATCA GTTACAAACCG	gprF deletion
gprF_LB_rev	CCTCCACTAGCATTACACTTAGTTAATGAGG GAGTTAGGTG	gprF deletion
gprF_hph_for	CGATCACCTAACTCCCTCATTAACTAAGTGT AATGCTAGTGGAGG	gprF deletion
gprF_hph_rev	CGCAGCCTTTATCAGGTCGGAATATTGGGG GGAGTTTAGGGAAA	gprF deletion
gprF_RB_for	CTTTCCCTAAACTCCCCCCAATATTCCGACC TGATAAAGGC	gprF deletion
gprF_RB_rev	ATTGTAGGAGATCTTCTAGAAAGATCAATTG GCTAACTTATAGGTCT	gprF deletion
gprF_orf_for	ATGGATAACCCCGTTGCCG	Analytic PCR
gprF_orf_rev	TACCTCAATTTCATTACTTCTCC	Analytic PCR
gprF_ex_for	CATATTCTTGGACGAGACGAG	Analytic PCR
gprF_ex_rev	TCTCCCGCATGAATAAGGAAG	Analytic PCR
gprA_q_for	ACTGGGATATTCGCGAGTCAA	qRT-PCR
gprA_q_rev	AAAGGATGCCTCGGAGAATAAC	qRT-PCR
gprB_q_for	CCGATCCTATCGTGCTCGAT	qRT-PCR
gprB_q_rev	TCCAGACAATCTCCTGATCCG	qRT-PCR
gprC_q_for	GCCATTGCAATCGTAACGGTC	qRT-PCR
gprC_q_rev	AGGCTCGGCTTTAATCCAGCA	qRT-PCR
gprD_q_for	CGTCATCGCCATCGCATTAAT	qRT-PCR
gprD_q_rev	GTCGACCCTTTCCGTCCAA	qRT-PCR
gprE_q_for	GCCAGACCGAGCTTTCTTCA	qRT-PCR
gprE_q_rev	CTCGTTGCTTCAATCGTAACTG	qRT-PCR
gprF_q_for	CAGCTTATCCCTCAGATAGTG	qRT-PCR

gprF_q_rev	GCCCATGTAAGCAGACTCAAG	qRT-PCR
H2b-FW	GAAGAAGGCAGGAAAGAAGAC	qRT-PCR
H2b-Rev	TTTGGCAGACGAGGAAGAG	qRT-PCR
BD-GasA-for	ATATGGCCATGGAGGCCGAATTCCCATGGG TTGCAGCATGTCTAC	Y2H
BD-GasA-rev	GGCCGCTGCAGGTCGACGGATCCCCTTATA TCAGACCGCAGTTTCTG	Y2H
AD-GasA-for	CAACGCAGAGTGGCCATTATGGCCCATGGG TTGCAGCATGTCTAC	Y2H
AD-GasA-rev	CGAGGCGGCCGACATGTTTTTTCCCTTATAT CAGACCGCAGTTTCTG	Y2H
gprC_adhP_ol_for	AGCTATACCAAGCATACAATCAACTATGGCC TTCACGACACTTTC	Y2H
gprC_AD_ol_rev	CTTTATCCATCTTTGCAAAGCTTGGCTCTCT TCGCGGTCCCG	Y2H
gprC_BD_ol_r-n	TTTCAGGAGGCTTGCTTCAAGCTTGGCTCTC TTCGCGGTCCCG	Y2H
gprCtail_adhP_ol_for	AGCTATACCAAGCATACAATCAACTAACGAA AGAGTCTGGAGACAA	Y2H
gprCtail_AD_ol_rev	CTTTATCCATCTTTGCAAAGCTTGGCTCTCT TCGCGGTCCC	Y2H
gprCtail_BD_ol_rev	TTCAGGAGGCTTGCTTCAAGCTTGGCTCTCT TCGCGGTCCC	Y2H
AD_C_for	CCAAGCTTTGCAAAGATGGAT	Y2H Backbone
adhP_rev	AGTTGATTGTATGCTTGGTATAG	Y2H Backbone
BD_C_for	CCAAGCTTGAAGCAAGCCT	Y2H Backbone
