# Supplementary material for: GprC of the nematode-trapping fungus Arthrobotrys flagrans activates mitochondria and reprograms fungal cells for nematode hunting
Source: Nat Microbiol. 2024 Jun 14;9(7):1752–63. doi: 10.1038/s41564-024-01731-9 (PMC11222155; doi:10.1038/s41564-024-01731-9)
Supplement: Supplementary file 2 — Reporting Summary [file 41564_2024_1731_MOESM2_ESM.pdf]

nature portfolio

Corresponding author(s): Reinhard fischer

Last updated by author(s): May 3, 2024

Reporting Summary

Nature Portfolio wishes to improve the reproducibility of the work that we publish. This form provides structure for consistency and transparency in reporting. For further information on Nature Portfolio policies, see our Editorial Policies and the Editorial Policy Checklist.

Statistics

For all statistical analyses, confirm that the following items are present in the figure legend, table legend, main text, or Methods section.

n/a	Confirmed	
☐	☒	The exact sample size (*n*) for each experimental group/condition, given as a discrete number and unit of measurement
☐	☒	A statement on whether measurements were taken from distinct samples or whether the same sample was measured repeatedly
☐	☒	The statistical test(s) used AND whether they are one- or two-sided<br>*Only common tests should be described solely by name; describe more complex techniques in the Methods section.*
☒	☐	A description of all covariates tested
☒	☐	A description of any assumptions or corrections, such as tests of normality and adjustment for multiple comparisons
☐	☒	A full description of the statistical parameters including central tendency (e.g. means) or other basic estimates (e.g. regression coefficient) AND variation (e.g. standard deviation) or associated estimates of uncertainty (e.g. confidence intervals)
☐	☒	For null hypothesis testing, the test statistic (e.g. *F*, *t*, *r*) with confidence intervals, effect sizes, degrees of freedom and *P* value noted<br>*Give P values as exact values whenever suitable.*
☒	☐	For Bayesian analysis, information on the choice of priors and Markov chain Monte Carlo settings
☒	☐	For hierarchical and complex designs, identification of the appropriate level for tests and full reporting of outcomes
☒	☐	Estimates of effect sizes (e.g. Cohen's *d*, Pearson's *r*), indicating how they were calculated

Our web collection on statistics for biologists contains articles on many of the points above.

Software and code

Policy information about availability of computer code

Data collection	Microscopic images were collected by ZEN 2012 Blue Edition and AxioVision software. Seahorse XFe24 was operated for measurement of oxygen consumption rate. Fiji/ImageJ 2.0 was used for CTCF quantification. CFX Connect Real-Time PCR Detection System was used for qPCR data collection.
Data analysis	Phyre2 and TMHMM 2.0 were used for transmembrane helices prediction. The protein structures were predicted by AlphaFold 2.3. Autodock Vina 1.2.3 was used for protein-ligand docking. Alignments and illustrations were performed with VMD 1.9.4. Ligands were built with Avogadro 1.2.0 and geometry optimized with Orca 5.0.1. Statistics were analyzed by GraphPad Prism 8.0. The signal peptide and mitochondrial targeting sequence were predicted by ipsort and mitoprot.

For manuscripts utilizing custom algorithms or software that are central to the research but not yet described in published literature, software must be made available to editors and reviewers. We strongly encourage code deposition in a community repository (e.g. GitHub). See the Nature Portfolio guidelines for submitting code & software for further information.

Data

Policy information about availability of data

All manuscripts must include a data availability statement. This statement should provide the following information, where applicable:

- Accession codes, unique identifiers, or web links for publicly available datasets
- A description of any restrictions on data availability
- For clinical datasets or third party data, please ensure that the statement adheres to our policy

All data generated or analyzed during this study are included in this published article or source data file. Source data are provided with this paper. The Duddingtonia (Arthrobotrys) flagrans genome database used in this study is from National Center for Biotechnology Information GenBank under the accession number PRJNA494930. References to this accession number can be found throughout this paper.

Research involving human participants, their data, or biological material

Policy information about studies with human participants or human data. See also policy information about sex, gender (identity/presentation), and sexual orientation and race, ethnicity and racism.

Reporting on sex and gender	N/A
Reporting on race, ethnicity, or other socially relevant groupings	N/A
Population characteristics	N/A
Recruitment	N/A
Ethics oversight	N/A

Note that full information on the approval of the study protocol must also be provided in the manuscript.

Field-specific reporting

Please select the one below that is the best fit for your research. If you are not sure, read the appropriate sections before making your selection.

☒ Life sciences ☐ Behavioural & social sciences ☐ Ecological, evolutionary & environmental sciences

For a reference copy of the document with all sections, see nature.com/documents/nr-reporting-summary-flat.pdf

Life sciences study design

All studies must disclose on these points even when the disclosure is negative.

Sample size	The determination of sample sizes was based on the previous experiments (https://doi.org/10.1038/s41467-021-25535-1) in order to ensure the statistical reproducibility.
Data exclusions	No data were excluded from the analyses.
Replication	All of the experiments were performed by at least three biological and technical repeats independently. Data shown in graphs or plots represent mean ± the standard deviation (SD) and Plotted data points are shown. The statistical significance is considered as the $P < 0.05$. All attempts were successful.
Randomization	The samples were allocated randomly into wild type and gene knock-out mutants.
Blinding	Blinding is not relevant to the experiments in this paper. For all of the experiments, the measurements and analyses were performed identically in all conditions. This paper is mainly for the characterization of molecular and genetic features of Arthrobotrys flagrans.

Reporting for specific materials, systems and methods

We require information from authors about some types of materials, experimental systems and methods used in many studies. Here, indicate whether each material, system or method listed is relevant to your study. If you are not sure if a list item applies to your research, read the appropriate section before selecting a response.

Materials & experimental systems

n/a	Involved in the study
☐	☒ Antibodies
☒	☐ Eukaryotic cell lines
☒	☐ Palaeontology and archaeology
☐	☒ Animals and other organisms
☒	☐ Clinical data
☒	☐ Dual use research of concern
☒	☐ Plants

Methods

n/a	Involved in the study
☒	☐ ChIP-seq
☒	☐ Flow cytometry
☒	☐ MRI-based neuroimaging

Antibodies

Antibodies used

anti-GFP antibody(11814460001; Roche);
anti-phospho-p38 MAP kinase (Tyr180/Tyr182) antibody (#9211; Cell Signaling Technology);
anti-phospho-p44/42 MAPK (Erk1/2) (Thr202/Tyr204) antibody (#9101; Cell Signaling Technology);
anti-Histone H3 antibody (#ab1791; abcam);
anti-rabbit IgG (whole molecular)-peroxidase antibody (A0545; Sigma-Aldrich);
anti-mouse IgG (Fab specific)-Peroxidase antibody (A2304; Sigma-Aldrich)

Validation

These antibodies are commercially available and have been extensively validated. Anti-GFP antibody is applicable for IP, WB and IF (https://www.sigmaaldrich.com/DE/de/product/roche/11814460001?utm_source=google&utm_medium=cpc&utm_campaign=12414022935&utm_content=117752994226&gclid=CjwKCAjw88yxBhBWEiwA7cm6pdFFdpCmZ9RxpRry2Zv7eHJbmBDIGC0N_TTW4lvB2xrUAy8kFzYz0BoCWGYQAvD_BwE#product-documentation). The anti-phospho-p38 MAP kinase (Tyr180/Tyr182) antibody is validated for IP, WB and IF in species including human, mouse, zebrafish, S.cerevisiae(https://www.cellsignal.com/products/primary-antibodies/phospho-p38-mapk-thr180-tyr182-antibody/9211). The anti-phospho-p44/42 MAPK (Erk1/2) (Thr202/Tyr204) antibody is validated for WB, IP, IF, IHC, CHIP, C&R, C&T, DB and F in species including human, mouse, zebrafish, S.cerevisiae (https://www.cellsignal.com/products/primary-antibodies/phospho-p44-42-mapk-erk1-2-thr202-tyr204-antibody/9101). The anti-Histone H3 antibody is applicable for WB, IHC-P, CHIP, ICC/IF and IP in species including human, mouse, zebrafish, Neurospora crassa(https://www.abcam.com/en-de/products/primary-antibodies/histone-h3-antibody-nuclear-marker-and-chip-grade-ab1791#).

Animals and other research organisms

Policy information about studies involving animals; ARRIVE guidelines recommended for reporting animal research, and Sex and Gender in Research

Laboratory animals

Caenorhabditis elegans N2 strain was used. And the mixed-age (including all the larva and adults) was used in this paper.

Wild animals

This study did not involve wild animals.

Reporting on sex

The sex in the C. elegans group was not considered.

Field-collected samples

The study did not use field-collected samples.

Ethics oversight

It is not necessary for invertebrates.

Note that full information on the approval of the study protocol must also be provided in the manuscript.

Plants

Seed stocks

N/A

Novel plant genotypes

N/A

Authentication

N/A
