# Supplementary material for: GprC of the nematode-trapping fungus Arthrobotrys flagrans activates mitochondria and reprograms fungal cells for nematode hunting
Source: Nat Microbiol. 2024 Jun 14;9(7):1752–63. doi: 10.1038/s41564-024-01731-9 (PMC11222155; doi:10.1038/s41564-024-01731-9)

Fig. 2c

GprC-GFP

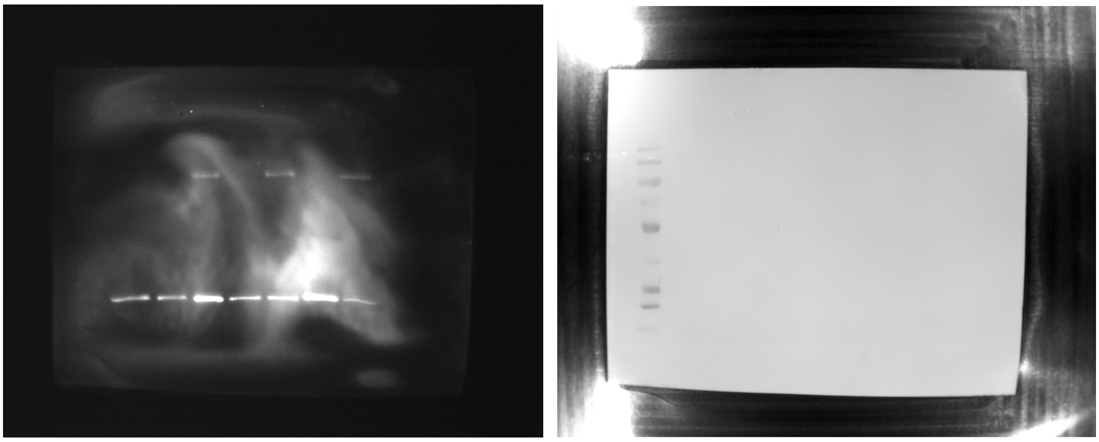

CitA-GFP

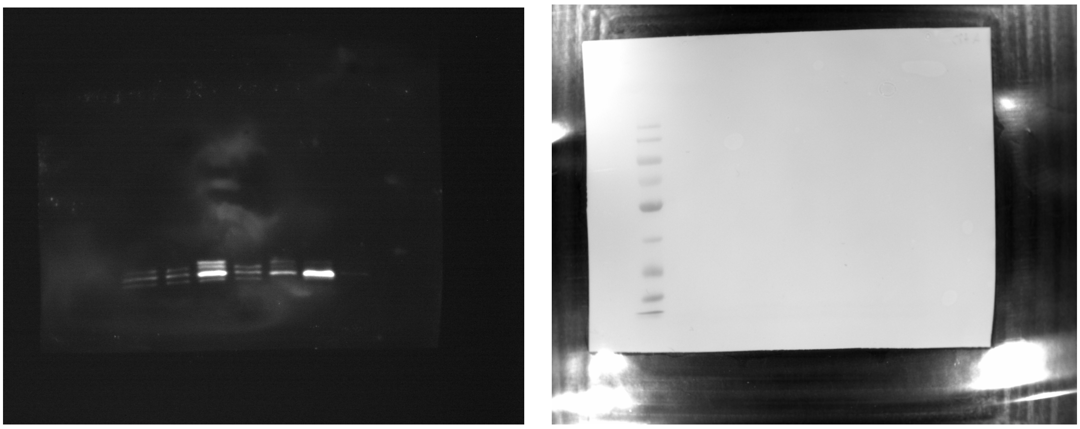

WT

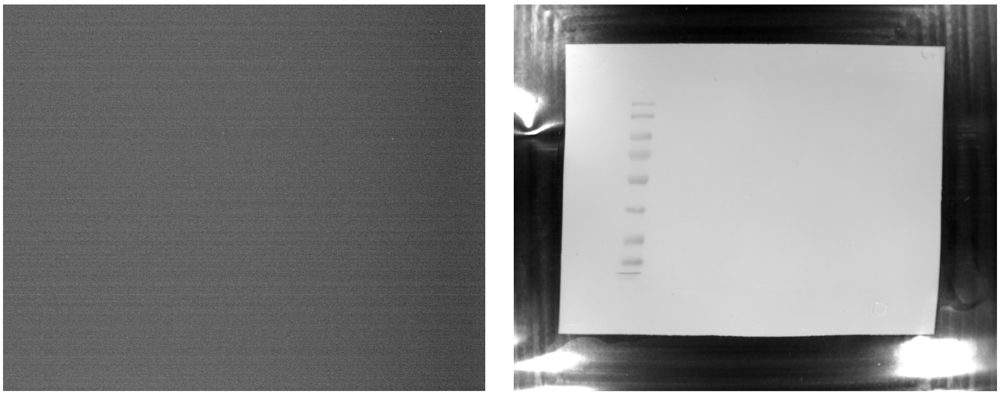

Fig. 3b

Anti-phospho-p44/42

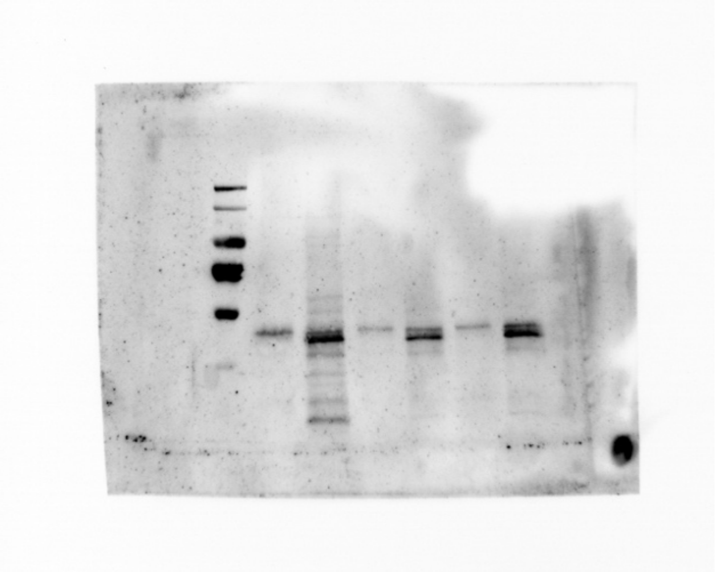

Anti-phospho-p38

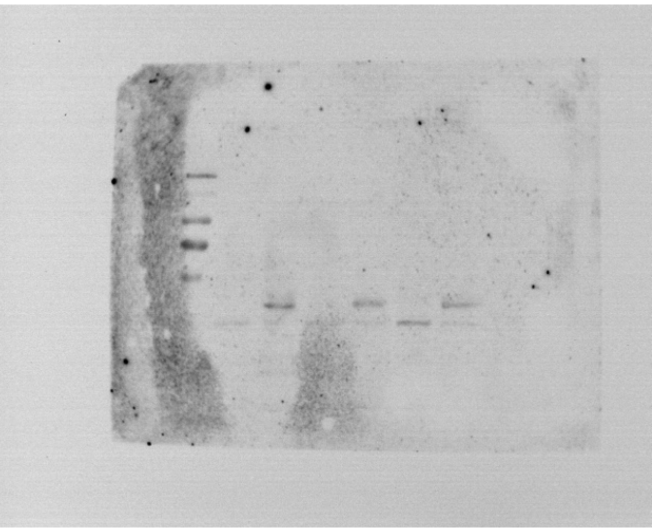

Anti-Histone H3

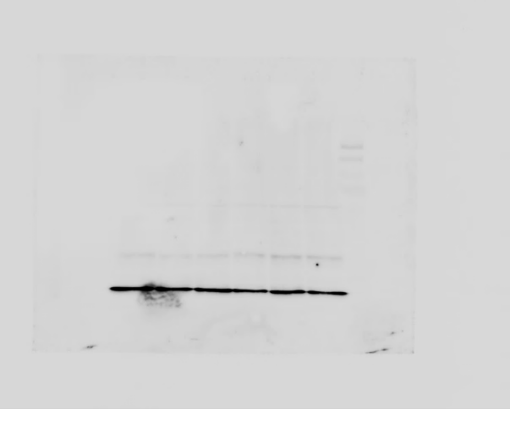

Extended Data Fig. 3

GprC-GFP

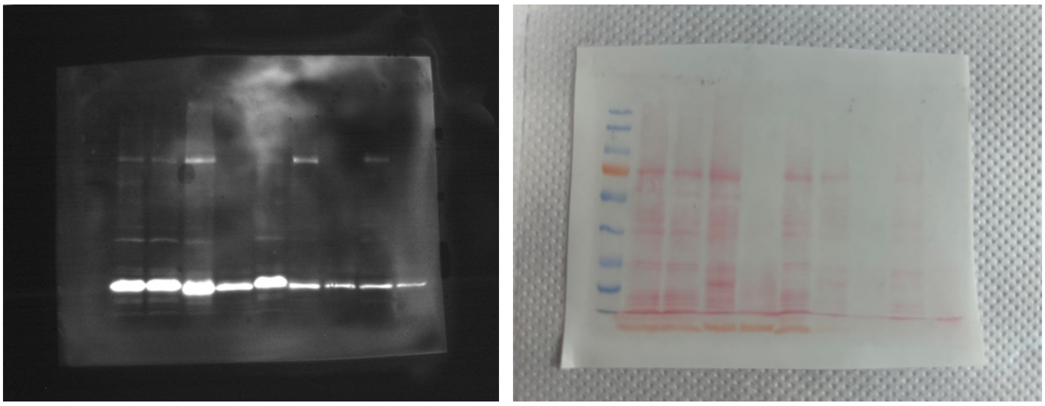

CitA-GFP

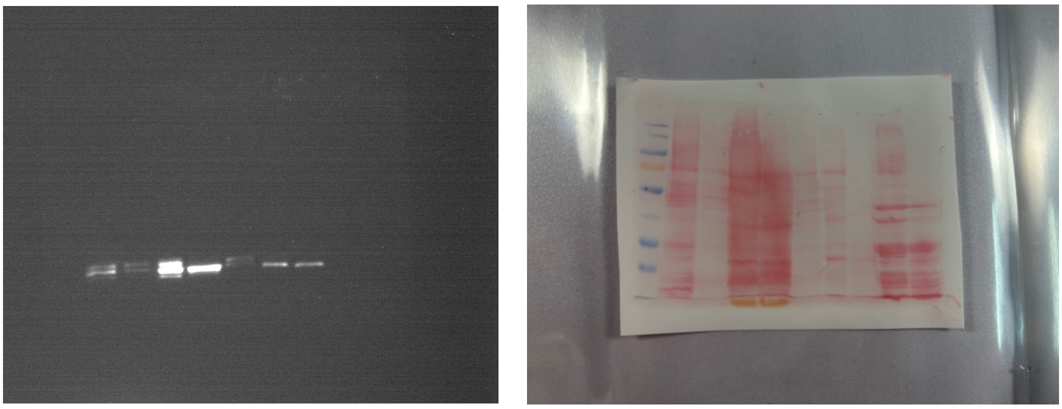

Supplement: Supplementary file 4 — Source data western blots. [file 41564_2024_1731_MOESM4_ESM.pdf]
