# Supplementary material for: GprC of the nematode-trapping fungus Arthrobotrys flagrans activates mitochondria and reprograms fungal cells for nematode hunting
Source: Nat Microbiol. 2024 Jun 14;9(7):1752–63. doi: 10.1038/s41564-024-01731-9 (PMC11222155; doi:10.1038/s41564-024-01731-9)

Extended Data Figure 1b

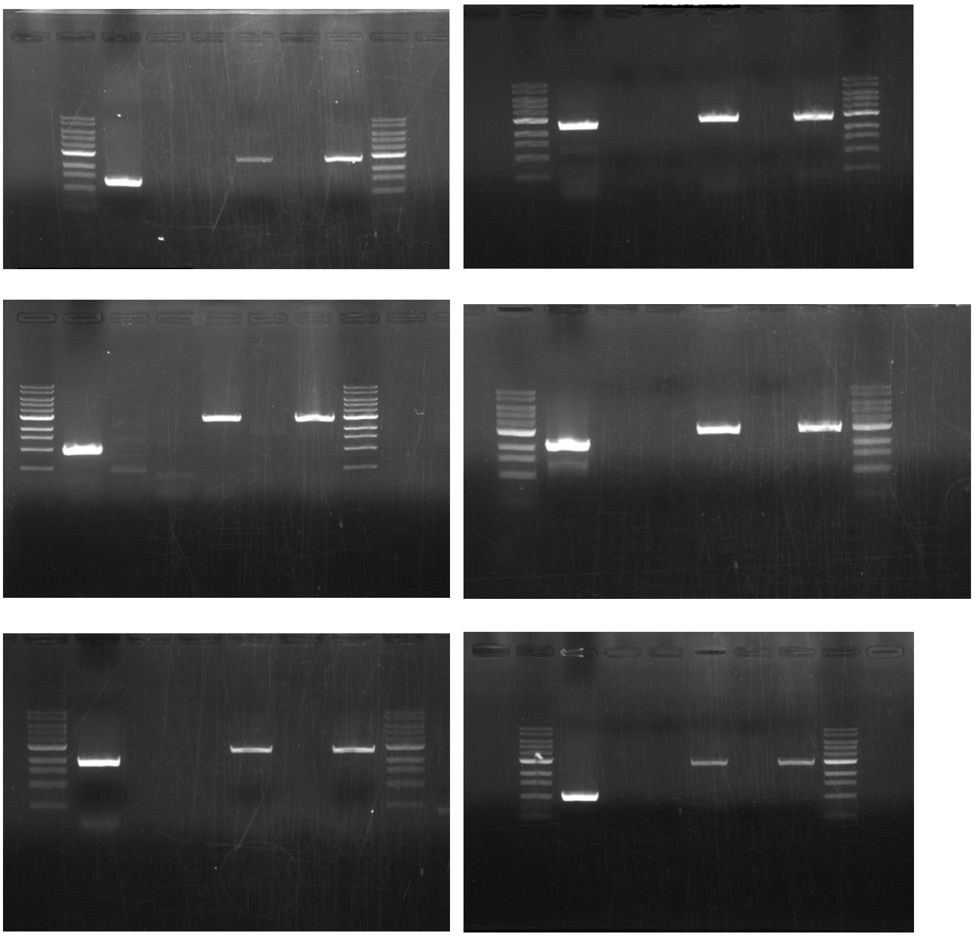

Extended Data Figure 1c

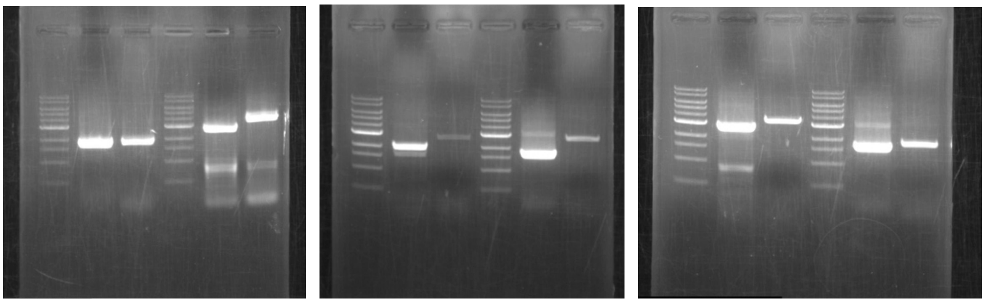

Extended Data Figure 1e

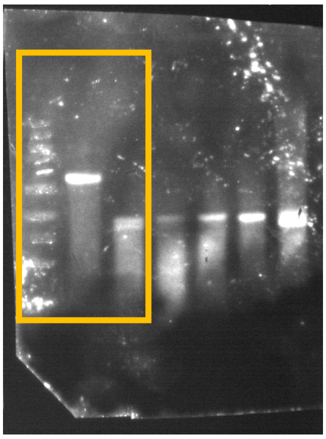

Extended Data Figure 2b

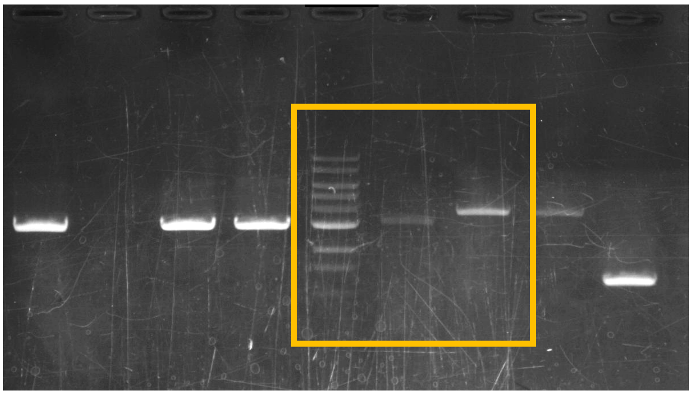

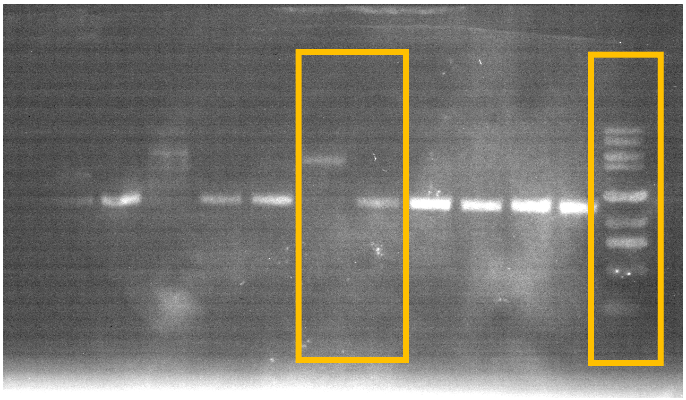

Extended Data Figure 2d

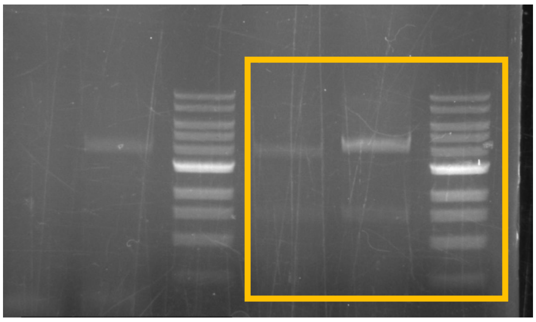

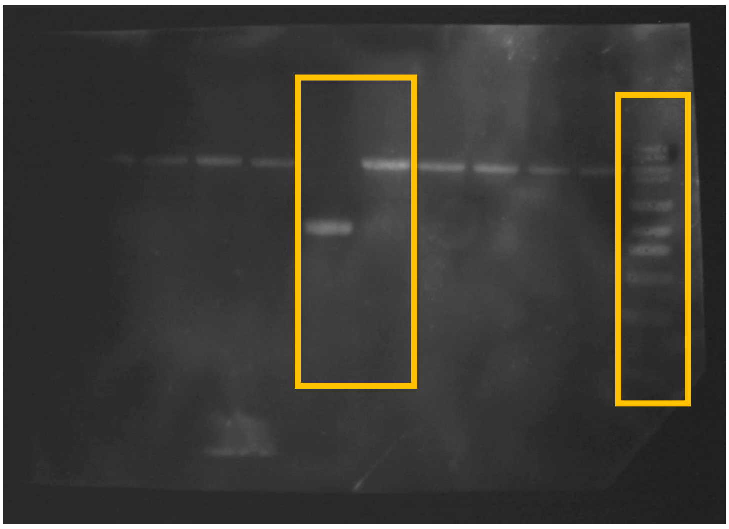

Extended Data Figure 2f

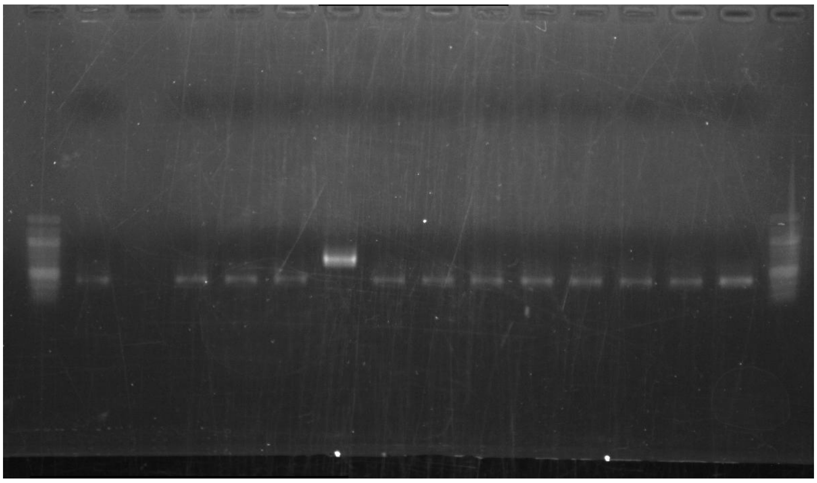

Supplement: Supplementary file 5 — Source data PCR and Southern blots. [file 41564_2024_1731_MOESM5_ESM.pdf]
